# A multi-scale transformer-enhanced YOLO framework for unified road damage detection and boundary-aware segmentation

**DOI:** 10.3389/frai.2026.1834179

**Published:** 2026-05-28

**Authors:** Bakhytzhan Kulambayev, Olzhas Olzhayev, Azizah Suliman

**Affiliations:** 1 Turan University, Almaty, Kazakhstan; 2 International Information Technology University, Almaty, Kazakhstan; 3 Asia Metropolitan University, Cyberjaya, Malaysia

**Keywords:** intelligent transportation systems, multi-scale feature extraction, road cracks, road damage detection, segmentation, transformers

## Abstract

Road surface deterioration poses significant challenges for transportation safety, infrastructure maintenance, and intelligent transportation management. Automated road damage analysis based on computer vision has therefore become an important research topic in recent years. This study proposes a novel deep learning framework for multi-task road damage analysis that integrates detection, classification, and segmentation within a unified architecture. The proposed model incorporates multi-scale feature extraction, transformer-based contextual refinement, ROI-based classification enhancement, and a boundary-aware segmentation module to improve the identification of diverse pavement defects. Multi-scale alignment enables the network to capture road damage patterns at different spatial resolutions, while contextual attention mechanisms strengthen feature relationships across complex road scenes. The boundary-aware segmentation branch further improves structural understanding of pavement defects by enabling precise contour delineation. Extensive experiments demonstrate that the proposed architecture achieves superior performance compared with conventional detection models, improving both detection accuracy and segmentation quality while maintaining practical inference speed. Ablation studies further confirm the effectiveness of each architectural component and highlight the contribution of contextual feature refinement and boundary-aware learning to overall model performance. The results indicate that the proposed framework provides a reliable and efficient solution for automated road condition monitoring and intelligent infrastructure inspection. This approach can support real-time road maintenance systems and contribute to safer and more efficient transportation networks.

## 1 Introduction

Road infrastructure constitutes a critical backbone of modern transportation systems, directly influencing economic activity, public safety, and long-term urban sustainability. However, pavement surfaces are continuously exposed to mechanical stress, environmental degradation, and fluctuating traffic loads, which collectively accelerate the formation of defects such as potholes, longitudinal cracks, transverse cracks, and alligator cracking. Manual inspection remains labor-intensive, subjective, and often inconsistent, particularly when deployed at large scale across metropolitan networks (Arya et al., 2021). As a result, automated vision-based road damage analysis has emerged as an essential research direction within intelligent transportation systems and smart city initiatives (Silva et al., 2023).

Early computer vision approaches relied on handcrafted texture descriptors and threshold-based segmentation techniques, which were highly sensitive to illumination variations and surface artifacts (Khan et al., 2024). With the advent of deep learning, convolutional neural networks (CNNs) significantly improved robustness by learning hierarchical spatial representations directly from data (Omarov, 2025). One-stage detectors such as YOLO introduced real-time performance capabilities, making large-scale deployment feasible for vehicle-mounted inspection systems (Ren et al., 2023). Nevertheless, conventional CNN-based detectors often struggle with thin, low-contrast crack structures and scale variability, especially when small defects coexist with larger surface anomalies (Wan et al., 2023).

Transformer architectures have recently demonstrated superior capacity for modeling long-range dependencies and global contextual relationships in visual recognition tasks (Mishra and Lourenço, 2024). Vision Transformers and hybrid CNN–Transformer frameworks have shown particular promise in multi-scale feature interaction and semantic reasoning (Iparraguirre et al., 2022). Integrating such contextual modeling mechanisms into object detection pipelines has improved performance in dense prediction tasks, yet the adaptation to road damage scenarios remains insufficiently explored (Deepa and Sivasangari, 2023). Furthermore, unified frameworks capable of jointly performing detection and boundary-aware segmentation are still limited, despite the practical importance of precise contour delineation for maintenance planning and severity assessment (Cha et al., 2025).

In this paper, we propose a Multi-Scale Transformer-Enhanced YOLO Framework for Unified Road Damage Detection and Boundary-Aware Segmentation. The core contribution lies in a transformer-based context refinement module that facilitates cross-scale feature interaction while preserving fine-grained spatial cues. Unlike conventional neck structures that rely solely on convolutional aggregation, the proposed architecture introduces global attention mechanisms to enhance semantic coherence across pyramid levels. Simultaneously, a decoupled multi-task prediction head integrates bounding box regression, classification refinement, and boundary-aware mask generation within a single coherent pipeline.

By bridging multi-scale convolutional representation learning with transformer-driven contextual modeling, the proposed framework aims to achieve robust detection accuracy while maintaining structural precision in crack segmentation. The resulting system is designed not merely for benchmark performance but for practical deployment in intelligent road monitoring environments, where reliability, scalability, and interpretability are paramount.

## 2 Related works

Automated road damage analysis has evolved considerably over the past decade, transitioning from handcrafted image processing pipelines to end-to-end deep learning frameworks. Early approaches relied heavily on edge detection, morphological filtering, and texture descriptors such as Gabor filters and local binary patterns to isolate crack-like structures (Kulambayev et al., 2022). Although these techniques demonstrated feasibility under controlled conditions, they exhibited limited generalization in the presence of shadows, oil stains, and complex pavement textures (Zeng and Zhong, 2024).

The introduction of convolutional neural networks marked a decisive shift toward data-driven feature extraction, enabling more robust crack and pothole detection under diverse environmental conditions (Wang and Cha, 2025). Fully convolutional networks and encoder–decoder architectures such as U-Net became particularly influential for pixel-level crack segmentation tasks due to their ability to preserve fine spatial detail through skip connections (Zhang and Liu, 2024). Subsequent enhancements incorporated attention gates and residual connections to mitigate vanishing gradients and enhance feature discrimination in low-contrast scenarios (Jiang et al., 2025; Elghaish et al., 2022).

Object detection frameworks further expanded the scope of automated pavement monitoring. Two-stage detectors, including Faster R-CNN variants, achieved high localization accuracy but incurred substantial computational cost, limiting real-time applicability (Fan et al., 2023). In contrast, one-stage detectors such as YOLO and SSD provided a favorable trade-off between speed and accuracy, making them suitable for vehicle-mounted inspection systems (Lingxin et al., 2022). Later iterations of YOLO introduced architectural refinements, including cross-stage partial connections and feature pyramid integration, improving multi-scale object representation (Chen et al., 2023). Nevertheless, these detectors often exhibited reduced sensitivity to slender crack patterns, particularly when cracks appeared fragmented or partially occluded (Wan et al., 2023).

To address multi-scale challenges, feature pyramid networks were widely adopted to aggregate hierarchical representations across different resolutions (Le-Xuan et al., 2024). Path aggregation networks and bidirectional feature fusion modules further enhanced cross-scale information flow, strengthening detection of small or subtle defects (Xiao et al., 2026). Despite these improvements, convolution-based fusion mechanisms primarily capture local spatial correlations, limiting their capacity to model long-range contextual dependencies across large receptive fields (Xie, 2026).

Transformer architectures have recently emerged as powerful alternatives for global feature modeling in computer vision tasks. Vision Transformer demonstrated that self-attention mechanisms could effectively replace or complement convolutional operations in image classification (Mishra and Lourenço, 2024). Hybrid CNN–Transformer models subsequently combined the locality of convolution with the global reasoning ability of self-attention, yielding improvements in segmentation and detection benchmarks (Teng et al., 2023). Multi-scale transformer modules have shown particular promise in dense prediction tasks, where interactions between coarse semantic features and fine spatial details are essential (Yamane et al., 2023).

In the context of road damage analysis, transformer-based models remain relatively nascent. Some studies incorporated attention-based modules into crack segmentation networks to enhance feature weighting and spatial relevance (Bragança et al., 2026). Others introduced transformer encoders within detection backbones to improve robustness against illumination variability and texture complexity (Raushan et al., 2025). However, many existing implementations focus exclusively on either detection or segmentation, rather than designing unified frameworks capable of performing both tasks concurrently (Martucci et al., 2023).

Boundary-aware segmentation has attracted increasing attention due to its importance in accurately delineating thin crack contours. Edge supervision strategies and auxiliary boundary losses have been employed to sharpen segmentation outputs and reduce over-smoothing effects in convolutional decoders (Jeong et al., 2022). Multi-task learning paradigms integrating detection and segmentation have demonstrated synergistic improvements, as shared representations encourage complementary feature learning across tasks (Zhang et al., 2024). Nevertheless, balancing multi-task loss functions remains a nontrivial optimization challenge, often requiring careful weighting strategies to prevent dominance of a single objective (Honarjoo et al., 2026).

Recent research trends emphasize the integration of contextual reasoning with multi-task detection frameworks to improve structural consistency in complex scenes (Abubakr et al., 2024). In transportation-related applications, such integration is particularly valuable, as accurate damage localization and contour delineation directly inform maintenance prioritization and cost estimation processes (Wang et al., 2023).

Building upon these developments, the proposed work advances the state of the art by combining multi-scale convolutional representation learning with transformer-based context refinement and boundary-aware segmentation within a unified YOLO-inspired architecture. Rather than treating detection and segmentation as isolated tasks, the framework explicitly models cross-scale interactions and integrates auxiliary boundary supervision to enhance structural precision. This design seeks to reconcile real-time efficiency with high-fidelity damage delineation, addressing limitations observed in both purely convolutional and standalone transformer-based approaches.

## 3 Materials and methods

This section presents the methodological foundation of the proposed Multi-Scale Transformer-Enhanced YOLO framework, detailing the architectural design, data preparation procedures, and optimization strategy adopted for unified road damage detection and boundary-aware segmentation. The framework is structured as a sequential yet tightly integrated pipeline comprising multi-scale feature extraction, transformer-based contextual refinement, multi-task prediction heads, and coordinated loss optimization. Each component is formally described to ensure reproducibility, while the overall formulation emphasizes the interaction between hierarchical representations and global attention mechanisms to address scale variability and structural complexity in real-world road inspection scenarios.

### 3.1 Problem formulation

Road surface monitoring requires accurate localization of structural defects together with precise delineation of their spatial extent. In real-world scenarios, damages such as cracks and potholes exhibit complex shapes, scale variations, and ambiguous textures, which make joint detection and segmentation a challenging vision problem.

**Figure 1:**
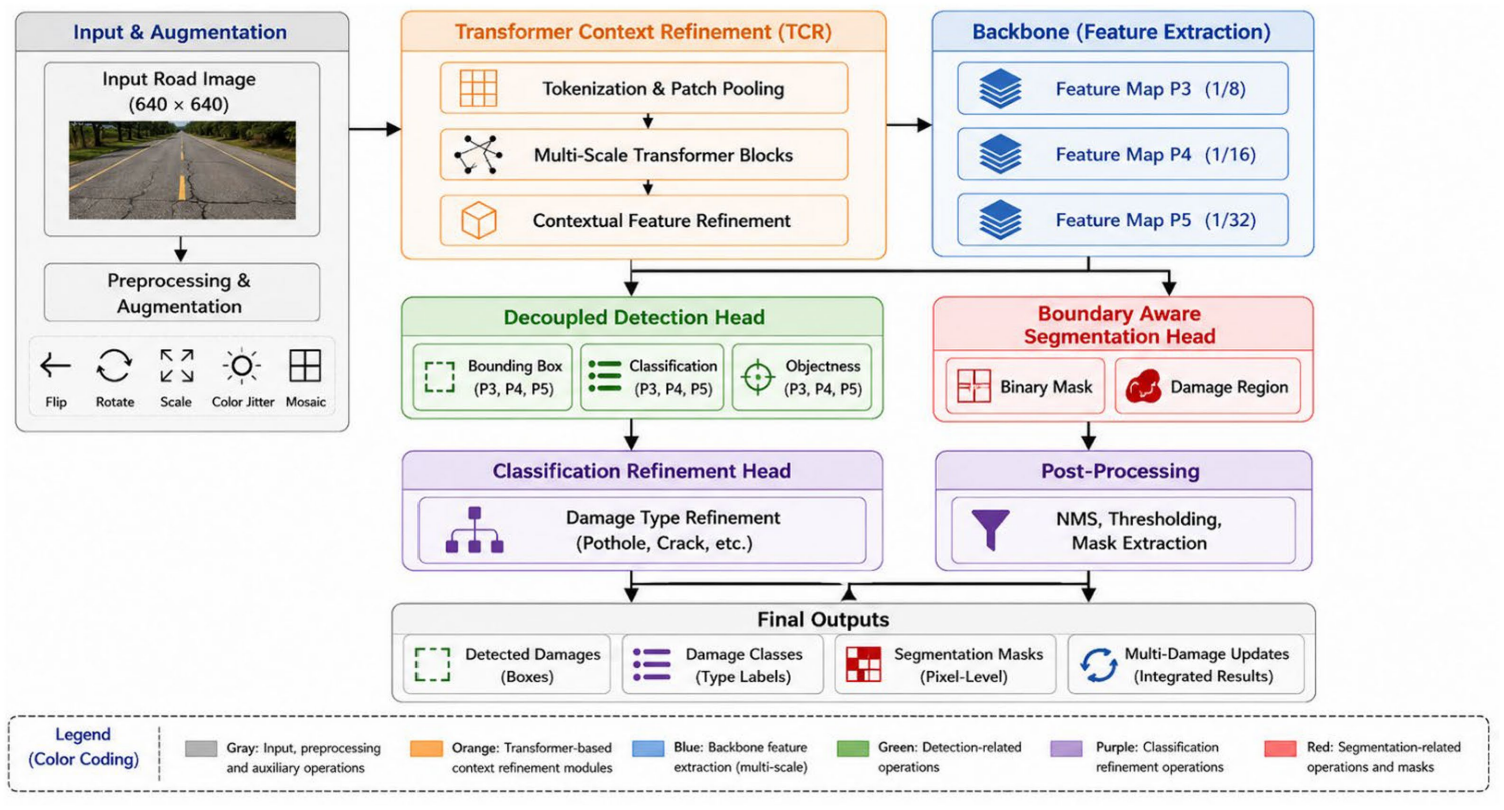
Overall architecture of the proposed multi-task road damage analysis framework integrating detection, classification, and segmentation branches. Consistent color coding is adopted throughout the architecture to improve interpretability and visual consistency. Blue denotes multi-scale feature extraction modules and backbone representations; orange represents transformer-based contextual refinement components; green corresponds to detection-related operations, including bounding box prediction and objectness estimation; purple indicates classification refinement and damage category prediction modules; red denotes segmentation-related operations and pixel-level mask generation; and gray represents preprocessing, augmentation, and auxiliary operations.

Figure 1 presents the high-level workflow of the proposed TCR-YOLO framework, illustrating how the input image is processed through multi-scale feature extraction, transformer-based refinement, and multi-task prediction heads to produce bounding boxes, class labels, and boundary-aware segmentation masks in a unified end-to-end manner. Let an input road image be denoted by Equation (1), while the unified multi-task prediction mapping is formulated in Equation (2).


$$I\in R^{H\times W\times 3}$$ (1)

Where H=W = 640 after resizing.

The proposed framework performs unified multi-task prediction through an end-to-end mapping function:


$$f_{\theta }:X\to (B,S,M,E)$$ (2)

Where $f_{\theta }$ denotes the complete multi-task deep neural network parameterized by learnable parameters θ , rather than an individual prediction branch. The function jointly maps the input image $X\in {\mathbb{R}}^{H\times W\times 3}$ into four output spaces corresponding to detection, classification, segmentation, and boundary prediction tasks.

### 3.2 Input preprocessing and augmentation

The input image is resized and normalized before being forwarded to the backbone, as formulated in Equation (3). We denote the resizing operator as R(·) and normalization as N(·):


$$X=N(R(I))\in R^{640\times 640\times 3}$$ (3)

During training, stochastic augmentation A(·) is applied as defined in Equation (4).


$$\tilde{X}=A(X)$$ (4)

This improves robustness to illumination shifts, motion blur, and background texture variations typical of real road scenes.

### 3.3 Multi-scale feature extraction and alignment

Accurate road damage analysis requires extracting discriminative features that simultaneously preserve fine-grained crack structures and encode broader contextual cues related to surrounding pavement conditions. Since defects such as longitudinal cracks, transverse cracks, and potholes appear at different spatial scales and exhibit substantial intra-class variability, a single-resolution representation is insufficient. Therefore, the proposed framework adopts a hierarchical feature extraction strategy that captures both low-level texture details and high-level semantic abstractions. This design enables robust representation learning across varying object sizes, illumination conditions, and background complexities commonly encountered in real-world road inspection scenarios.

**Figure 2:**
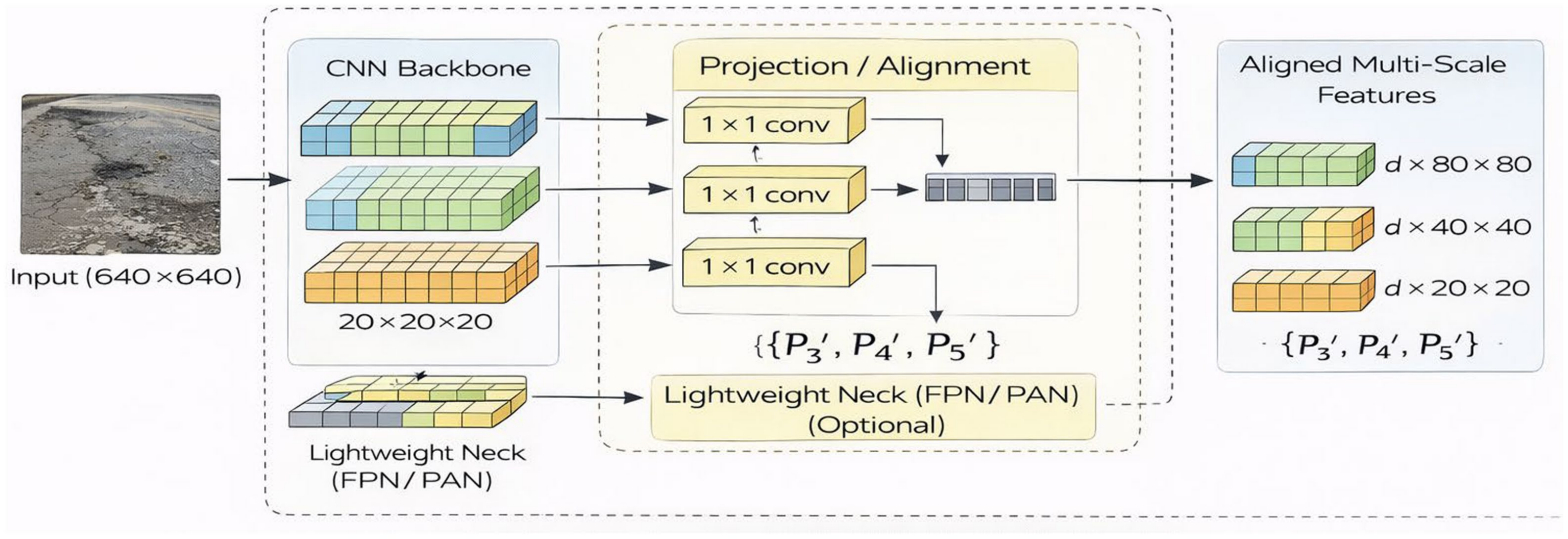
Multi-scale feature extraction and alignment.

Figure 2 details the multi-scale feature extraction stage composed of a CNN backbone and a projection-alignment module. The backbone generates a hierarchical feature pyramid as formulated in Equations (5)–(7) at three spatial resolutions, enabling the representation of both fine-grained crack patterns and larger structural defects. These multi-level features are subsequently projected into a unified embedding space to facilitate consistent cross-scale interaction in the subsequent transformer refinement stage:


$$P_{k}=\phi_{k}(\tilde{X}),k\in \{3,,4,,5\}$$ (5)

Where $P_{3}\in R^{C_{3\times 80\times 80}}$ , $P_{4}\in R^{C_{4\times 40\times 40}}$ , and $P_{5}\in R^{C_{5\times 20\times 20}}$ to standardize channel dimensionality prior to transformer-based fusion, each pyramid level is projected to a shared embedding dimension 𝑑 using 1 × 1 convolutions:


$$P_{k}^{\prime }=\mathrm{Con}v_{1\times 1}^{\left(k\right)}(P_{k})\in R^{d\times H_{k}\times W_{k}}$$ (6)

Optionally, a lightweight neck structure such as FPN or PAN may be incorporated to enhance bidirectional information flow across pyramid levels while maintaining computational efficiency. This component facilitates top-down and bottom-up feature aggregation, reinforcing semantic consistency between coarse and fine resolutions. When employed, the neck operation can be formally represented in an abstract functional form as follows.


$$\left\{P_{3}^{\prime },P_{4}^{\prime },P_{5}^{\prime }\}=\Psi_{\text{neck}}(P_{3},P_{4},P_{5}\right)$$ (7)

The aligned multi-scale feature pyramid $\{P_{3}^{\prime },P_{4}^{\prime },P_{5}^{\prime }\}\;$ is subsequently propagated to the Transformer Context Refinement module. At this stage, features are already normalized in channel dimensionality, enabling consistent tokenization and cross-scale interaction. This transition establishes the foundation for global contextual modeling beyond purely convolutional aggregation.

### 3.4 Transformer context refinement module

Enhancing contextual reasoning beyond local convolutional receptive fields requires mechanisms capable of modeling long-range dependencies across multiple spatial scales. A transformer-based refinement module is therefore incorporated to facilitate global feature interaction within the hierarchical pyramid structure. Purely convolutional fusion strategies are inherently constrained by localized receptive fields, which can restrict effective communication between distant yet semantically correlated pavement regions, particularly when defects are fragmented or spatially dispersed.

**Figure 3:**
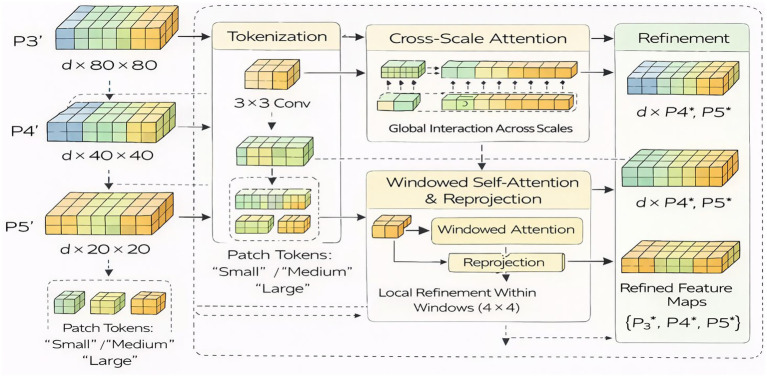
Transformer context refinement module.

Figure 3 illustrates the internal workflow of this refinement module in detail. As depicted, the aligned multi-scale feature maps are first transformed into token embeddings through patch-based projection, after which cross-scale attention is applied to facilitate information exchange among different resolutions. The refined tokens are then reprojected into spatial feature maps, preserving both contextual coherence and localized crack patterns. This process allows the network to jointly reason about thin fracture structures and broader surface context, improving robustness under complex visual conditions.

#### 3.4.1 Tokenization and patch pooling

Each aligned feature map $P_{k}^{\prime }$ is converted into a set of tokens through the tokenization process defined in Equations (8) and (9) via patch pooling or strided embedding. Let $\tau_{k}(\cdot )$ denote tokenization:


$$Z_{k}=\tau_{k}(P_{k}^{\prime })\in R^{N_{k}\times d}$$ (8)

Where $N_{k}$ is the number of tokens at scale k. A simple formulation uses spatial flattening after a local embedding layer:


$$Z_{k}=\text{Flatten}(\mathrm{Con}v_{3\times 3}(P_{k}^{\prime }))$$ (9)

The multi-scale token set is denoted by $Z=\{Z_{3},Z_{4},Z_{5.}\}$

#### 3.4.2 Cross-scale attention for global interaction

To explicitly model interactions across pyramid levels, we apply cross-scale attention between tokens. The cross-scale attention mechanism is mathematically formulated in Equations (10)–(14). For a query token matrix 𝑄, key matrix 𝐾, and value matrix 𝑉, scaled dot-product attention is defined as:


$$\text{Attn}(Q,K,V)=\text{Softmax}(\frac{QK^{T}}{\sqrt{d}})V$$ (10)

In cross-scale mode, queries may come from one scale while keys and values aggregate tokens from multiple scales, e.g.,


$$Z_{4}^{\mathrm{CS}}=\text{Attn}(Z_{4}W_{Q},[Z_{3},Z_{4},Z_{5}]W_{k},[Z_{3},Z_{4},Z_{5}]W_{V})$$ (11)

Where [⋅;⋅] denotes concatenation and $W_{Q},W_{K},W_{K}\in R^{d\times d}$ , are learned projections. Residual learning and normalization stabilize training:


$${\hat{Z}}_{k}=\mathrm{LN}(Z_{k}+Z_{k}^{\mathrm{CS}})$$ (12)

#### 3.4.3 Windowed self-attention and reprojection

To preserve locality and refine thin crack patterns, windowed self-attention is applied within spatial windows after reshaping tokens back to grids. Let W(·) partition tokens into windows; attention is computed per window 𝑤:


$$Z_{k}^{W}=\text{Attn}(Z_{k}^{w}W_{Q},Z_{k}^{w}W_{Q}W_{k},Z_{k}^{w}W_{Q}W_{V})$$ (13)

The refined tokens are then reprojected to multi-scale feature maps:


$$P_{k}^{*}=R_{k}({\hat{Z}}_{k})\in R^{d\times H_{k}\times H_{k}};k\in \{3;4;5\}$$ (14)

The refined tokens are then reprojected to multi-scale feature maps:

The refined pyramid $\left\{P_{3}^{*};P_{4}^{*};P_{5}^{*}\right\}$ provides context-enriched representations for the downstream multi-task heads.

### 3.5 Multi-task prediction heads

The proposed framework adopts a unified multi-task prediction strategy in which detection, classification refinement, and segmentation are jointly optimized within a shared representation space. This design encourages complementary feature learning, allowing localization cues to inform segmentation accuracy while refined semantic classification enhances instance-level discrimination. By integrating these objectives within a single architecture, the model leverages shared contextual features while preserving task-specific specialization through dedicated branches.

**Figure 4:**
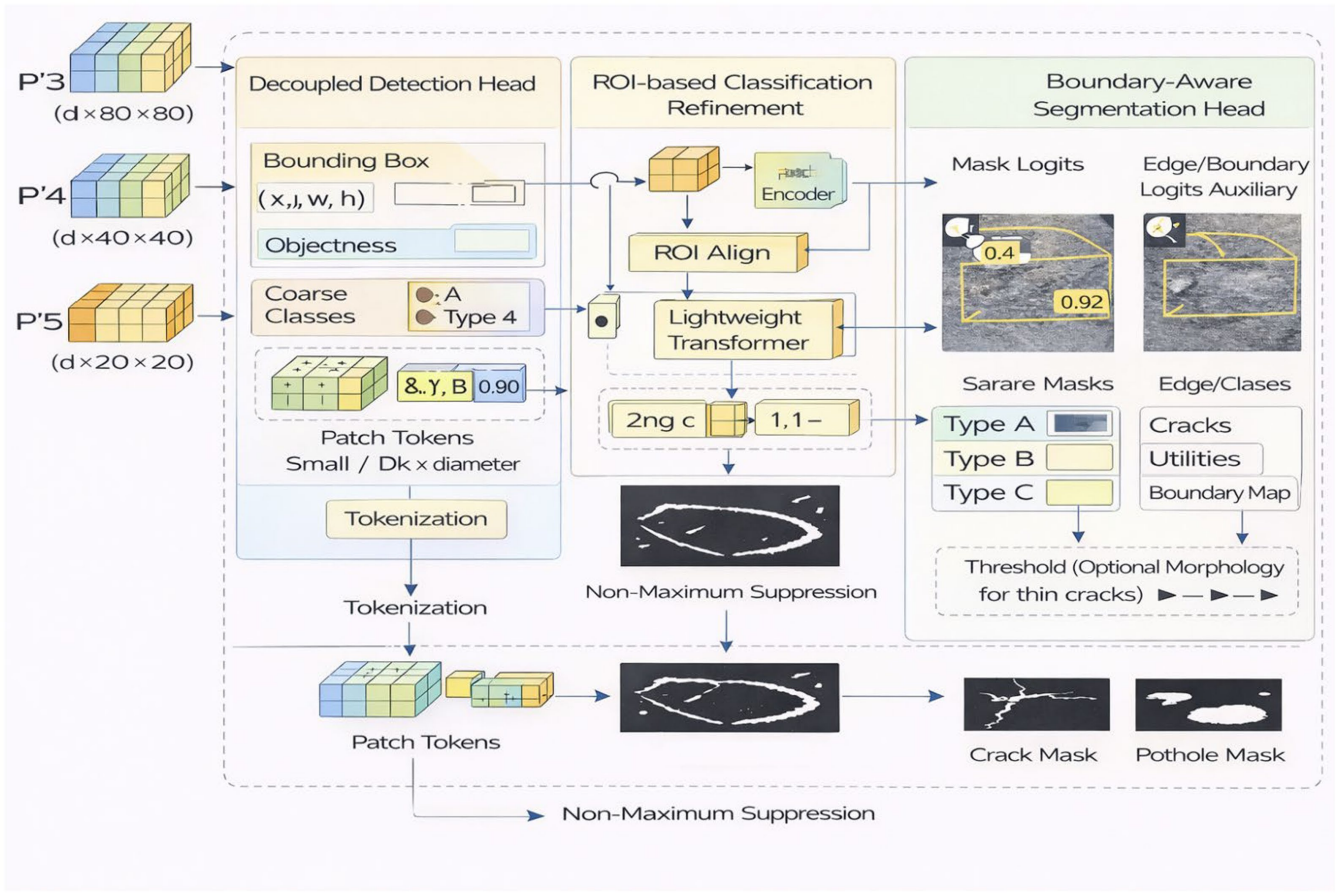
Multi-task prediction heads.

Figure 4 summarizes the three coupled prediction branches: a decoupled detection head responsible for bounding box regression and coarse classification, an ROI-based classification refinement module that revisits candidate regions for improved semantic separation, and a boundary-aware segmentation head that generates dense masks with explicit contour supervision. The figure illustrates how refined multi-scale features are distributed across these branches, enabling parallel yet coordinated prediction. This multi-branch formulation strengthens structural precision and semantic reliability, particularly in complex road scenes with overlapping or visually ambiguous defects.

#### 3.5.1 Decoupled detection head

The detection head predicts bounding box regression, objectness, and class probabilities as formulated in Equation (15). For a location 𝑖 on pyramid level 𝑘, the head outputs:


$$\left(t_{k,i},o_{k,i},c_{k,i})\;P_{k}^{*}=h_{\det}(Z_{k}^{*}\right)$$ (15)

Where $h_{k,i}\in R^{4}$ encodes box parameters, $o_{k,i}\in [0,1]$ is objectness, and $c_{k,i}\in R^{k}$ are class logits for 𝐾 damage categories. The full candidate set is obtained by aggregating predictions across k∈{3,4,5}.

#### 3.5.2 ROI-based classification refinement

Because damage categories can be visually similar under noise and wear, an ROI-based re$nement head revisits candidate detections. The ROI refinement and segmentation operations are defined in Equations (16) and (17), respectively. Given selected proposals $\hat{B}$ , ROI Align extracts aligned features:


$$R_{j}=\text{ROIAlign}(P^{*},{\hat{b}}_{j}),{\hat{b}}_{j}\in \hat{B}$$ (16)

Where $P^{*}$ denotes the fused set of refined pyramid maps and $R_{j}$ is an ROI tensor. A lightweight refinement network outputs corrected class logits $c_{j}^{\mathrm{ref}}$ , improving inter-class separation for cracks versus potholes under ambiguous textures.

#### 3.5.3 Boundary-aware segmentation head

The segmentation branch produces dense mask logits and an auxiliary boundary map, both derived from the refined pyramid. Let the segmentation head be $h_{\mathrm{seg}}$ :


$$\left(M,E)=h_{\mathrm{seg}}(P_{3}^{*},P_{4}^{*},P_{5}^{*}\right)$$ (17)

Where $M\in R^{640\times 640}$ and $M\in E^{640\times 640}$ represent mask and edge logits, respectively. Boundary supervision encourages sharper crack contours and reduces over-smoothing commonly observed in convolution-only decoders.

### 3.6 Training objective and optimization

The proposed training strategy adopts a coordinated multi-task optimization scheme that integrates detection, segmentation, boundary supervision, and classification refinement within a unified objective. Rather than optimizing each task independently, the framework enforces shared representation learning while preserving task-specific gradients through dedicated loss components. This design encourages complementary supervision, where localization accuracy supports segmentation quality and boundary awareness enhances structural coherence in crack delineation.

**Figure 5:**
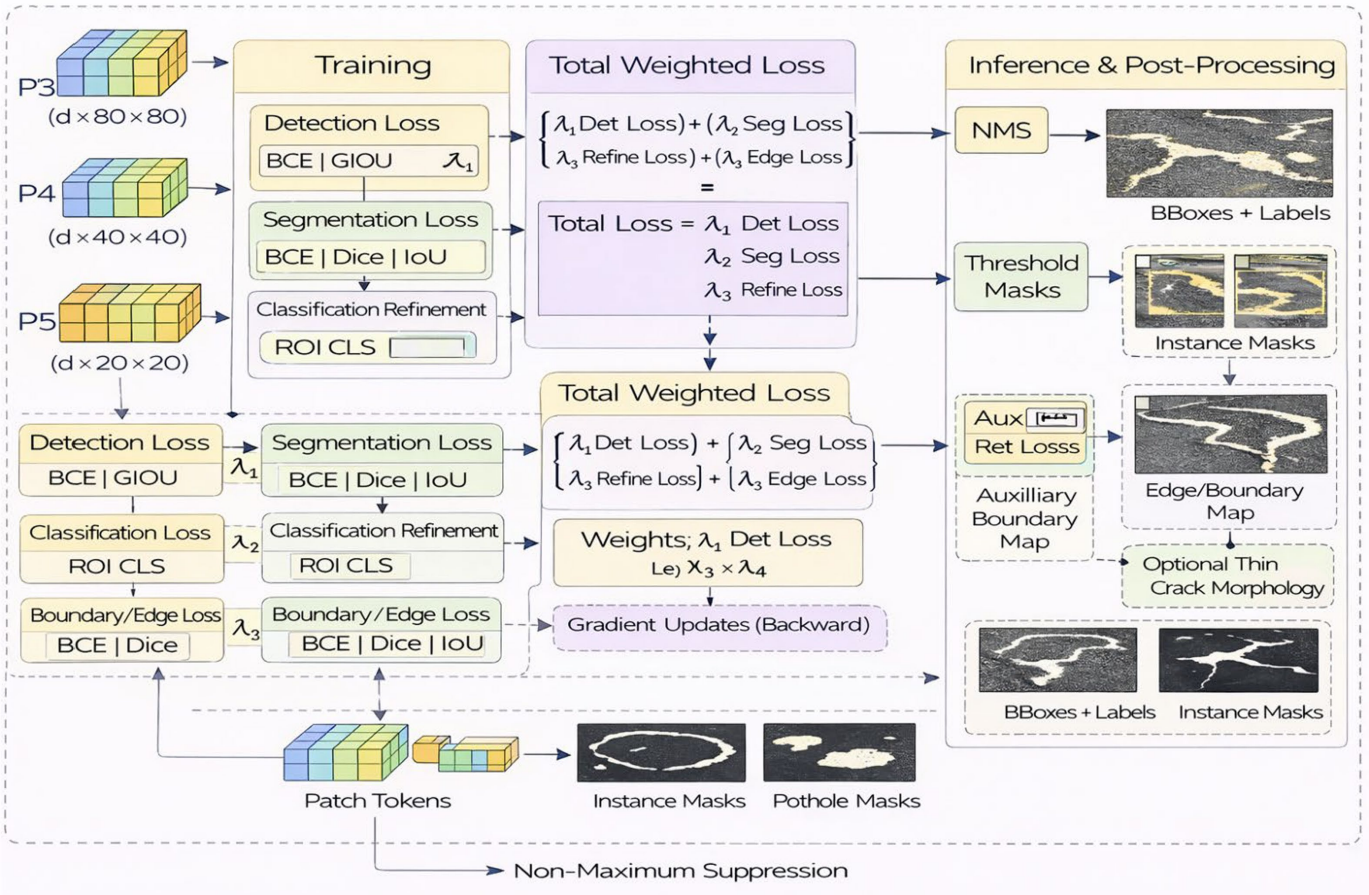
Decoupled detection head.

Figure 5 illustrates the overall training pipeline and the interaction among the different objective terms. The figure depicts how detection losses, segmentation loss, boundary supervision, and refinement classification loss are aggregated into a weighted total objective and propagated backward through the network. By visually summarizing the coupling between branches, Figure 5 clarifies how balanced multi-task supervision guides parameter updates, ensuring stable convergence and improved robustness across heterogeneous road damage patterns.

Let $L_{\det}$ denote the detection loss comprising classification, objectness, and regression terms formulated by Equation (18):


$$L_{\det}=L_{\det}+L_{\mathrm{obj}}+L_{\mathrm{box}}$$ (18)

For bounding box regression, a generalized IoU-style loss defined in Equation (19) is adopted to better capture geometric alignment between predicted and ground-truth boxes. This formulation penalizes not only overlap discrepancies but also spatial misalignment, thereby improving localization accuracy in complex road scenes.


$$L_{\mathrm{box}}=1-\text{GIoU}(b,b^{\mathrm{gt}})\;$$ (19)

The proposed framework adopts Generalized Intersection over Union (GIoU) loss for bounding box regression due to its stable optimization behavior under sparse and irregular road damage conditions. Unlike conventional IoU-based losses, GIoU introduces an additional geometric penalty term that remains effective even when predicted and ground-truth boxes do not overlap, which is particularly beneficial during early-stage optimization for elongated crack structures and small pavement defects. Although more recent alternatives such as Distance-IoU (DIoU) and Complete-IoU (CIoU) additionally incorporate center-distance and aspect-ratio constraints, preliminary experiments revealed only marginal localization improvements while slightly reducing optimization stability within the proposed multi-task learning framework. Therefore, GIoU was selected as a balanced compromise between localization accuracy, convergence robustness, and stable joint optimization across detection, segmentation, and classification objectives.

The segmentation and boundary supervision losses are formulated in Equations (20) and (21), respectively. In practice, a combination of Binary Cross-Entropy and Dice loss is employed to balance pixel-wise supervision with global structural alignment:


$$L_{\mathrm{seg}}=L_{\mathrm{BCE}}(\sigma (M),Y)\;$$ (20)

Where 𝑌 is the ground-truth mask and *σ*(·) is the sigmoid function. The boundary loss supervises edge logits against an edge target $Y_{e}$ .


$$L_{\text{edge}}=L_{\mathrm{BCE}}(\sigma (M),Y_{e})\;$$ (21)

The refinement classification objective is formulated in Equation (22). This focused supervision improves inter-class discrimination and reduces ambiguity between visually similar road damage categories. The refinement head is trained with a classification objective on ROI crops:


$$L_{\mathrm{ref}}=L_{\mathrm{CE}}(c^{\mathrm{ref}},y^{\mathrm{gt}})\;$$ (22)

To ensure balanced optimization across all learning objectives, the individual loss components are integrated into a unified formulation. The total multi-task optimization objective is formulated by Equation (23):


$$L_{\text{total}}=\lambda_{1}L_{\det}+\lambda_{2}L_{\mathrm{seg}}+\lambda_{3}L_{\text{edge}}+\lambda_{4}L_{\mathrm{ref}}$$ (23)

After defining the overall objective function, the network parameters are updated to minimize the aggregated loss. Model parameters are optimized via the gradient update rule defined in Equation (24)


$$\theta \leftarrow \theta -\eta \nabla_{\theta }L_{\text{total}}$$ (24)

Where 𝜂 is the learning rate.

The joint optimization of these objectives enables the model to balance localization accuracy, semantic discrimination, contour precision, and instance-level refinement within a single coherent learning framework. The weighting coefficients $\lambda_{1}$ , $\lambda_{2}$ , $\lambda_{3}$ , $\lambda_{4}$ are empirically tuned to ensure stable convergence and prevent dominance of any single task during training. This coordinated multi-task supervision promotes shared feature robustness while preserving task-specific sensitivity, ultimately enhancing both detection reliability and segmentation fidelity in complex road environments.

The weighting coefficients $\lambda_{1}$ , $\lambda_{2}$ , $\lambda_{3}$ , and $\lambda_{4}$ were determined through a controlled validation-based tuning procedure. Initially, all coefficients were set to 1.0 to establish a balanced baseline. Subsequently, a limited grid search was performed by varying one coefficient at a time within the range {0.25,0.50,1.00,1.50,2.00}, while keeping the remaining coefficients fixed. Each configuration was trained under identical optimization settings and evaluated on the validation set using mAP, F1-score, mIoU, boundary F1-score, and convergence stability. The final coefficient values were selected based on the best overall trade-off between detection accuracy, classification reliability, segmentation quality, and boundary preservation, rather than optimizing a single metric alone.

### 3.7 Inference and post-processing

During inference, the detector produces a set of candidate $\left\{(b_{i},s_{i})\right\}$ with scores $s_{i}$ derived from objectness and class confidence. Non-maximum suppression (NMS), which formulated by Equations (25)–(27), as shown in Figure 5, selects a consistent subset $B^{*}$ :


$$B^{*}=\mathrm{NMS}(\{b_{i},s_{i}\},\tau_{\mathrm{IoU}})\;$$ (25)

During inference, continuous mask logits are first transformed into probabilistic confidence maps. For segmentation, mask probabilities are obtained by *σ*(M) and thresholded:


$$\hat{Y}(u,v)=I(M(u,v))>\tau_{m}\;$$ (26)

Where I(⋅) is the indicator function and $\tau_{m}$ is a mask threshold. For thin cracks, optional morphology can be applied as a lightweight refinement step:


$$\hat{Y}=M_{\text{morph}}(\hat{Y})\;$$ (27)

The final outputs consist of refined detection boxes with associated damage labels, complemented by boundary-aware segmentation masks and explicit edge maps. This unified output structure allows the framework to simultaneously localize defects and delineate their precise contours. As a result, the system provides both instance-level interpretability and pixel-level structural accuracy within a single end-to-end pipeline.

### 3.8 Data collection and preparation

The dataset used in this study consists of a hybrid collection combining publicly available benchmark datasets and self-collected road inspection images captured in Kazakhstan urban and suburban environments. Public benchmark samples were obtained from datasets including RDD2022 and related road damage repositories, while additional vehicle-mounted camera recordings were collected from road networks in Almaty and nearby regions under varying environmental conditions, including daylight, nighttime, rain, and shadowed pavement scenarios.

**Figure 6:**
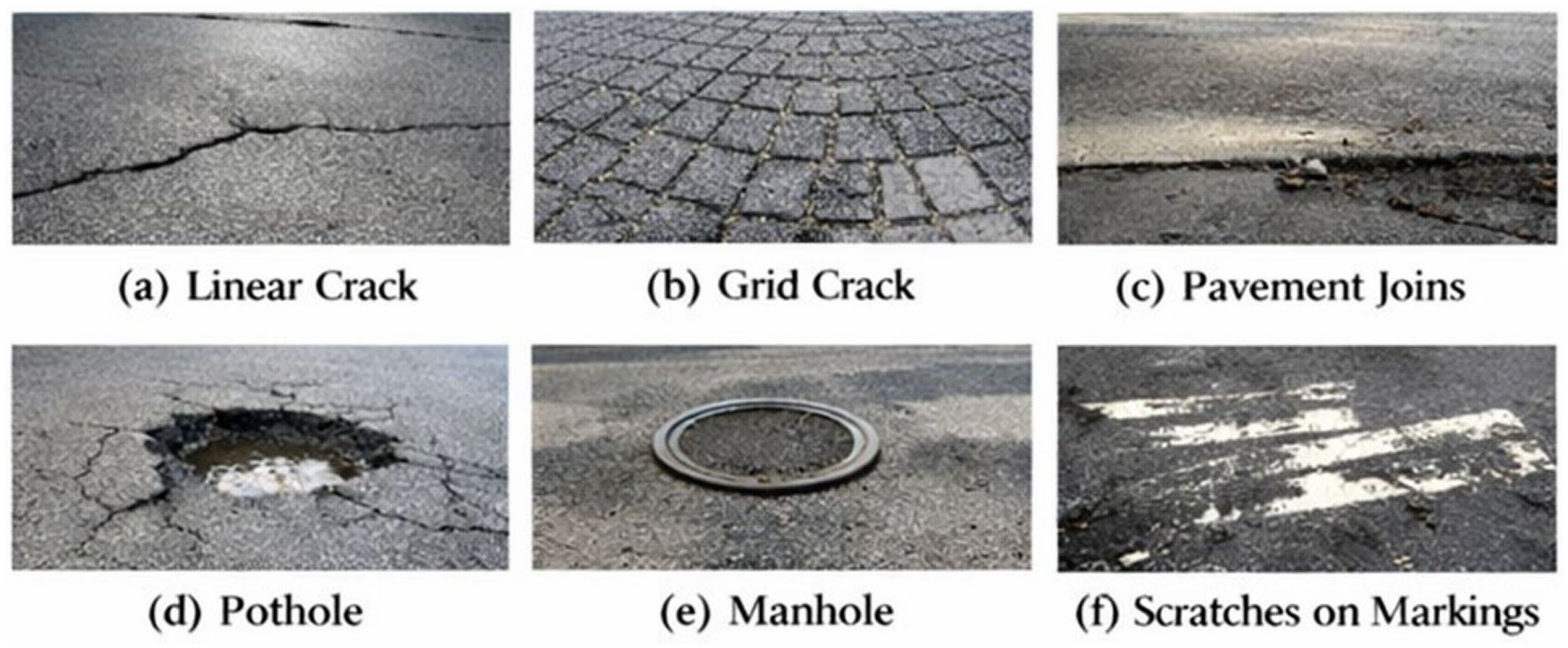
Representative samples of annotated road damage categories captured from vehicle-mounted cameras.

A comprehensive road surface dataset was curated to support unified detection and boundary-aware segmentation under realistic driving conditions. Image acquisition was performed using forward-facing vehicle-mounted cameras operating in urban and suburban environments, thereby capturing authentic road scenarios characterized by varying illumination, motion blur, occlusions, shadows, and perspective distortion. All images were standardized to a spatial resolution of 640 × 640 pixels to align with the input requirements of the proposed architecture. Figure 6 illustrates representative samples from the dataset, highlighting the diversity of crack morphologies, surface degradations, and contextual elements encountered during data collection.

The dataset comprises 12 annotated classes that encompass both structural road damages and visually confounding pavement elements. These include linear and grid cracks, repaired regions, patchings, potholes, manholes, stains, pavement markings, and other surface irregularities. As summarized in Table 1, the dataset contains a total of 19,440 annotated instances distributed across training, validation, and testing splits. Notably, certain categories such as “Scratches on markings” and “Linear crack” exhibit higher instance counts, whereas classes such as “Grid crack in patchings” and “Stains” are relatively less represented. This class imbalance reflects real-world frequency distributions and introduces a meaningful challenge for robust model generalization.

**Table 1:** Statistical distribution of annotated road damage instances across training, validation, and testing sets.

Class ID	Damage category	Training	Validation	Testing	Total
1	Linear crack	3,080	660	660	4,400
2	Grid crack	658	141	141	940
3	Pavement joins	854	183	183	1,220
4	Patchings	448	96	96	640
5	Fillings	1,344	288	288	1920
6	Potholes	406	87	87	580
7	Manholes	336	72	72	480
8	Stains	266	57	57	380
9	Shadow	1,190	255	255	1700
10	Pavement markings	1,414	303	303	2020
11	Scratches on markings	3,360	720	720	4,800
12	Grid Crack in patchings	252	54	54	360
—	Total	13,608	2,916	2,916	19,440

All images were manually annotated using a polygon-based segmentation protocol to ensure precise boundary delineation at the pixel level. Each visible instance within the primary drivable region of the image was segmented and assigned a corresponding class identifier. Bounding boxes were automatically derived from the annotated masks to facilitate joint supervision for detection and segmentation tasks. To preserve statistical consistency, the dataset was partitioned into training, validation, and testing subsets following a stratified sampling strategy, approximately maintaining a 70:15:15 ratio per class as reported in Table 1.

The 12-class taxonomy presented in Table 1 was designed based on a combination of publicly available road damage benchmark datasets, practical pavement inspection protocols, and real-world intelligent transportation requirements. The selected categories include both structural pavement defects and visually confounding environmental patterns frequently encountered in road monitoring applications. Structural damage classes such as linear cracks, grid cracks, potholes, patchings, fillings, pavement joins, and grid cracks in patchings were included because they directly reflect pavement deterioration severity and maintenance conditions commonly evaluated in road inspection systems.

In addition to structural defects, contextual and visually ambiguous categories including shadows, pavement markings, stains, and scratches on markings were intentionally incorporated to improve model robustness under realistic environmental conditions. These categories frequently introduce false detections in automated inspection systems due to strong visual similarity with actual pavement damage. Therefore, their inclusion enables the proposed framework to better distinguish true structural defects from environmental artifacts and non-damage pavement patterns. This taxonomy design aligns with practical intelligent transportation scenarios where road surfaces are affected by heterogeneous illumination, weather conditions, road markings, and urban infrastructure elements.

Prior to training, a structured preprocessing pipeline was applied, including normalization, geometric transformations, color jittering, and random spatial augmentations. These procedures increase variability in appearance and enhance generalization to unseen road conditions. Together, the dataset design, annotation rigor, and balanced partitioning establish a realistic and challenging benchmark for evaluating transformer-enhanced multi-task road damage analysis frameworks.

### 3.9 Implementation details

The proposed framework was implemented using the PyTorch 2.1 deep learning framework with CUDA 12.1 acceleration support. All experiments were conducted on a workstation equipped with an NVIDIA RTX 4090 GPU with 24 GB memory, an Intel Core i9 processor, and 64 GB RAM operating under Ubuntu 22.04 LTS. Model training was performed using the AdamW optimizer with an initial learning rate of $1\times 10^{-4}$ , weight decay of $1\times 10^{-5}$ , and cosine annealing learning rate scheduling. The network was trained for 100 epochs using a batch size of 16 with an input image resolution of 640×640 . Mixed precision training was employed to improve computational efficiency and reduce GPU memory consumption (Table 2).

**Table 2:** Experimental configuration and training environment.

Component	Configuration
Deep learning framework	PyTorch 2.1
CUDA version	CUDA 12.1
GPU	NVIDIA RTX 4090 (24 GB)
CPU	Intel Core i9
RAM	64 GB
Operating system	Ubuntu 22.04 LTS
Optimizer	AdamW
Initial learning rate	(1 \times 10^{−4})
Weight decay	(1 \times 10^{−5})
Learning rate scheduler	Cosine Annealing
Batch size	16
Input resolution	(640 \times 640)
Training epochs	100
Mixed precision training	Yes
Inference evaluation	Single-image batch
Average FPS	38

During inference evaluation, FPS measurements were obtained using single-image batch processing under fixed input resolution conditions without test-time augmentation. The reported inference speed corresponds to the average processing rate measured over the complete testing dataset. The proposed framework achieved real-time performance while simultaneously performing detection, classification, and segmentation tasks within a unified architecture.

In addition, the lightweight multi-scale CNN backbone and selective transformer refinement design improve computational efficiency compared with fully transformer-based architectures. This design characteristic makes the proposed framework potentially suitable for deployment on intelligent transportation edge devices and embedded GPU platforms such as NVIDIA Jetson systems. Future work will investigate model quantization, pruning, and lightweight optimization strategies for low-power real-time deployment scenarios.

Table 3 summarizes the training hyperparameters and implementation settings used for optimizing the proposed multi-task road damage analysis framework. The model was trained using the AdamW optimizer, which provides stable convergence and improved generalization through decoupled weight decay regularization. An initial learning rate of $1\times 10^{-4}$ together with cosine annealing scheduling enabled gradual and stable optimization throughout the 100 training epochs, reducing the risk of premature convergence and overfitting. The selected momentum parameters $\beta_{1}=0.9$ and $\beta_{2}=0.999$ ensured smooth gradient updates during multi-task learning. A batch size of 16 and input resolution of 640×640 were chosen to balance computational efficiency and feature representation quality. Mixed precision training further improved GPU memory utilization and accelerated training performance. In addition, extensive data augmentation was employed to improve robustness under varying road and environmental conditions. The inference evaluation performed using single-image batch processing demonstrates that the proposed framework achieves an average processing speed of 38 FPS, confirming its suitability for real-time intelligent transportation and road monitoring applications.

**Table 3:** Training hyperparameters.

Parameter	Value
Optimizer	AdamW
Initial learning rate	(1 \times 10^{−4})
Weight decay	(1 \times 10^{−5})
(\beta_1)	0.9
(\beta_2)	0.999
Learning rate scheduler	Cosine Annealing
Batch size	16
Input resolution	(640 \times 640)
Training epochs	100
Mixed precision training	Yes
Data augmentation	Yes
Inference evaluation	Single-image batch
Average FPS	38

## 4 Results

The experimental evaluation provides a comprehensive assessment of the proposed framework for automated road damage analysis. The model was tested using standard performance indicators such as accuracy, precision, recall, F1-score, and Intersection over Union in order to measure both detection reliability and segmentation quality across different defect categories. Quantitative results demonstrate the capability of the proposed architecture to accurately identify and localize various pavement defects under diverse road conditions. In addition to numerical evaluation, qualitative visualization results are presented to illustrate how the model detects and delineates structural damage patterns within complex road scenes. Comparative experiments and ablation studies are also conducted to investigate the influence of individual architectural components and to validate the effectiveness of the proposed design. The obtained results confirm that the developed framework achieves a strong balance between detection accuracy, segmentation precision, and computational efficiency, indicating its suitability for real-world road condition monitoring and intelligent infrastructure inspection systems.

### 4.1 Evaluation metrics

To evaluate classification and detection performance of the proposed framework, Accuracy, Precision, Recall, and F1-score were computed on the test set (Omarov et al., 2020). These metrics provide complementary insights into prediction reliability, class discrimina-tion capability, and error distribution across damage categories. The evaluation metrics used for quantitative performance assessment are formulated in Equations (28)–(31).

Accuracy measures the overall proportion of correctly predicted instances among all predictions and is defined as


$$\text{Accuracy}=\frac{\mathrm{TP}+\mathrm{TN}}{\mathrm{TP}+\mathrm{TN}+\mathrm{FP}+\mathrm{FN}}$$ (28)

Where TP denotes true positives, TN true negatives, FP false positives, and FN false negatives. While Accuracy provides a global performance indicator, it may be less informative under class imbalance conditions, which are common in road damage datasets.

Precision quantifies the correctness of positive predictions and is defined as


$$\text{Precision}=\frac{\mathrm{TP}}{\mathrm{TP}+\mathrm{FP}}$$ (29)

High Precision indicates that the model produces fewer false alarms, which is particularly important in practical road inspection systems where unnecessary maintenance actions must be avoided.

Recall evaluates the model’s ability to correctly identify all relevant damage instances and is expressed as:


$$\text{Recall}=\frac{\mathrm{TP}}{\mathrm{TP}+\mathrm{FN}}$$ (30)

A high Recall value implies effective recovery of actual defects, reducing the risk of missing critical structural damage.

The F1-score combines Precision and Recall into a single harmonic mean:


$$F1-\text{score}=2\cdot \frac{\text{precision}\cdot \text{recall}}{\text{precision}+\text{recall}}$$ (31)

This metric penalizes extreme imbalance between Precision and Recall and therefore provides a balanced measure of classification robustness. In multi-class evaluation, macro-averaged and weighted F1-scores were additionally computed to account for uneven class distributions across different types

Collectively, these evaluation metrics enable a comprehensive and balanced assessment of the proposed framework across detection, classification, and segmentation tasks. By jointly analyzing Accuracy, Precision, Recall, and F1-score, the evaluation captures not only overall correctness but also the trade-off between false positives and false negatives, which is critical in safety-sensitive road monitoring applications. This multi-metric strategy ensures that performance improvements are meaningful, robust, and practically relevant for real-world deployment scenarios.

### 4.2 Experiment results

This subsection presents the experimental evaluation of the proposed road damage analysis framework, focusing on both detection and segmentation performance across multiple defect categories. The experiments were conducted using the prepared dataset under controlled training and testing settings to assess the effectiveness of the proposed architecture. Quantitative metrics such as accuracy, precision, recall, F1-score, and Intersection over Union were employed to evaluate the model’s performance comprehensively (Hicks et al., 2019). In addition to the overall quantitative results, detailed analyses including confusion matrices, qualitative detection examples, and ablation studies are provided to better understand the contribution of individual architectural components. The results demonstrate the capability of the proposed model to accurately detect and delineate various types of road surface damage while maintaining practical computational efficiency suitable for real-world deployment scenarios.

**Figure 7:**
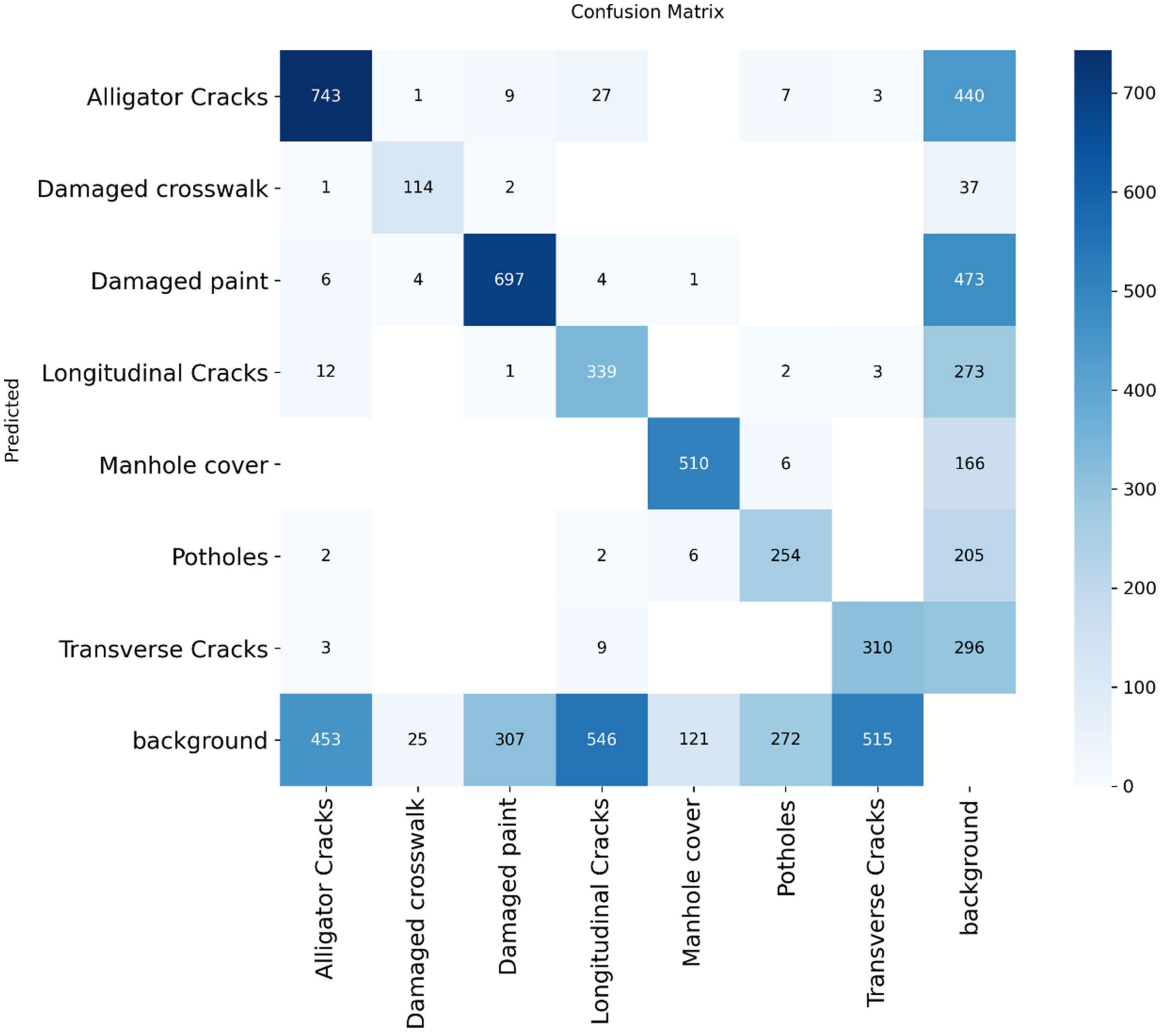
Confusion matrix illustrating classification performance of the proposed road damage detection model across seven damage categories and the background class.

Figure 7 illustrates the confusion matrix of the proposed road damage detection framework across seven damage categories and the background class. The diagonal values dominate most categories, indicating strong classification capability of the model. In particular, Alligator Cracks, Damaged paint, Manhole cover, and Transverse Cracks exhibit high true positive counts (743, 697, 510, and 310 respectively), demonstrating reliable feature discrimination for these defect types. The model also shows solid performance for Longitudinal Cracks and Potholes, with 339 and 254 correct predictions, although minor confusion with visually similar crack structures can be observed. For example, a small number of longitudinal cracks are misclassified as alligator cracks or transverse cracks, reflecting structural similarity between crack patterns.

Some misclassification is primarily associated with the background class, which absorbs difficult or ambiguous samples that contain weak or partially visible defects. A noticeable number of instances originally belonging to crack-related categories are predicted as background, particularly for Longitudinal Cracks and Transverse Cracks, suggesting that thin or low-contrast crack structures occasionally challenge the detector. Nevertheless, the overall matrix structure demonstrates that the proposed architecture maintains strong class separation with limited cross-category confusion. These results confirm the effectiveness of the multi-scale feature extraction and transformer-based contextual refinement components in capturing both global structural context and fine-grained damage characteristics.

**Figure 8:**
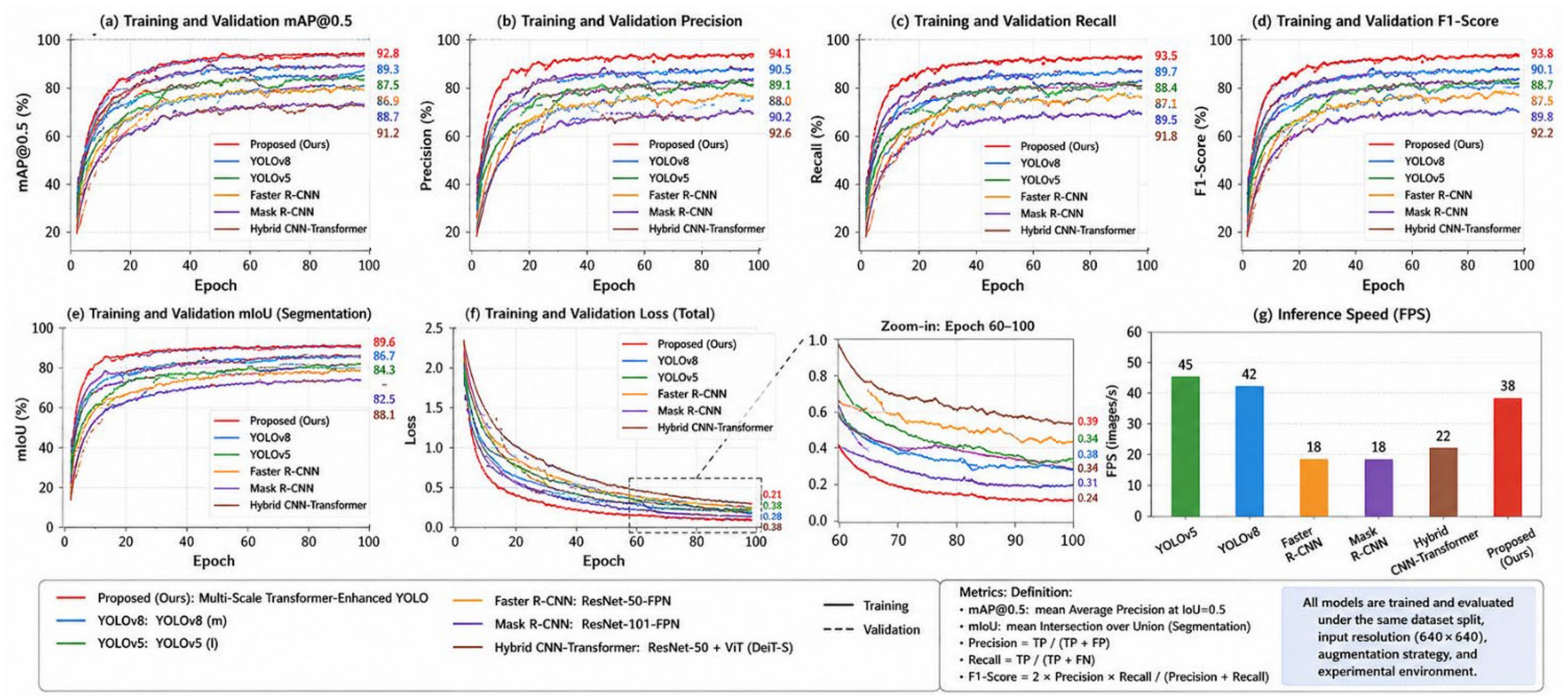
Performance analysis curves of the proposed road damage detection model.

Figure 8 presents the detailed training and validation behavior of the proposed framework together with several widely adopted baseline models across multiple evaluation metrics, including mAP@0.5, Precision, Recall, F1-score, mIoU, total loss convergence, and inference speed. As illustrated in Figures 8a–e, the proposed model consistently achieves the highest performance throughout the training process, ultimately reaching 92.8% mAP, 94.1% Precision, 93.5% Recall, 93.8% F1-score, and 89.6% mIoU. The convergence curves demonstrate that the proposed architecture learns more rapidly during early epochs and stabilizes with lower fluctuation compared to conventional CNN-based methods. In particular, the transformer-enhanced contextual refinement mechanism significantly improves feature representation capability, enabling more accurate localization and segmentation of fine-grained road defects such as thin longitudinal and transverse cracks. Furthermore, the relatively small gap between training and validation curves indicates strong generalization capability and reduced overfitting despite the complexity of the multi-task learning framework.

The loss convergence analysis shown in Figure 8f further confirms the optimization stability of the proposed model. The proposed framework achieves the lowest final loss value among all compared methods, indicating more effective feature learning and task balancing during joint optimization. The zoomed-in convergence region between epochs 60 and 100 demonstrates that the proposed architecture maintains smoother and more stable convergence behavior compared to hybrid and two-stage detection models, which exhibit larger fluctuations and slower stabilization. Although lightweight detectors such as YOLOv5 and YOLOv8 achieve slightly higher inference speed, the proposed model maintains a competitive real-time processing capability of 38 FPS while simultaneously performing detection, classification, and segmentation tasks. This balance between accuracy and computational efficiency highlights the effectiveness of integrating multi-scale CNN representations with transformer-based contextual reasoning. Overall, the results presented in Figure 8 demonstrate that the proposed framework provides superior robustness, segmentation quality, and detection reliability under diverse road inspection conditions.

**Figure 9:**
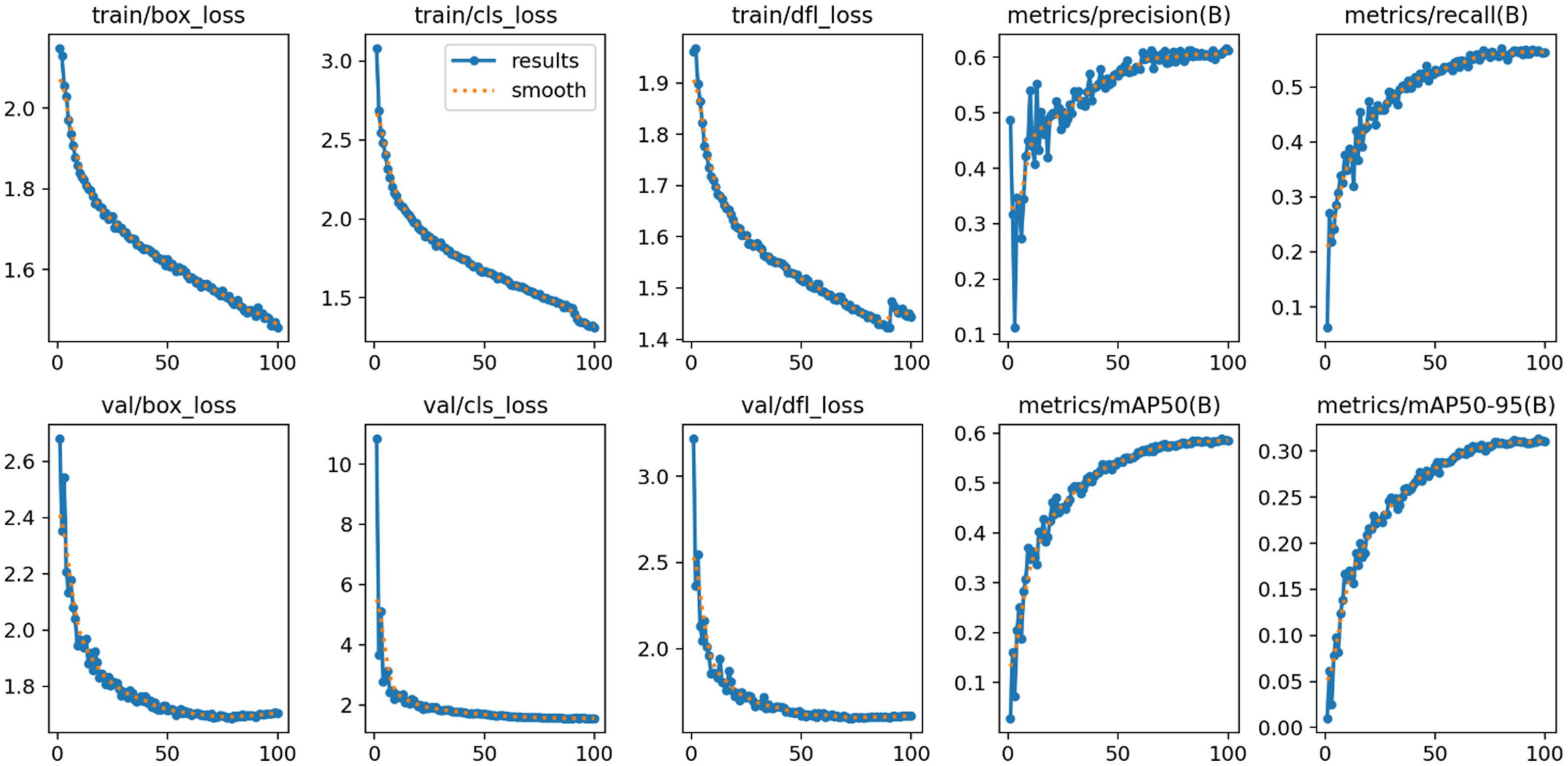
Training and validation performance curves of the proposed road damage detection model, illustrating loss convergence (box, classification, and DFL losses) and evaluation metrics including precision, recall, mAP@0.5, and mAP@0.5:0.95 across training epochs.

Figure 9 presents the training and validation dynamics of the proposed road damage detection model across 100 training epochs, including loss convergence and performance metrics. The training losses, namely box loss, classification loss, and distribution focal loss (DFL), demonstrate a consistent decreasing trend throughout the training process. Specifically, the box regression loss gradually decreases from approximately 2.1 to around 1.48, indicating progressive improvement in bounding box localization accuracy. Similarly, the classification loss shows stable convergence from roughly 3.0 to about 1.35, suggesting that the model effectively learns discriminative representations for different road damage categories. The DFL loss also decreases steadily, confirming improved confidence calibration in bounding box distribution prediction. Correspondingly, the validation losses follow a similar downward trajectory, which indicates good generalization capability and limited overfitting during training.

In addition to the loss convergence, the evaluation metrics illustrate progressive performance improvements over epochs. The precision metric steadily increases and stabilizes around 0.60, while recall gradually rises to approximately 0.56, reflecting improved detection reliability and coverage of damage instances. Furthermore, the mean Average Precision at IoU threshold 0.5 (mAP@0.5) increases consistently during training and converges near 0.59, demonstrating effective learning of object localization and classification simultaneously. The more stringent metric mAP@0.5:0.95 also improves steadily, reaching approximately 0.31 by the final training stage. These trends indicate stable optimization and balanced learning across detection tasks, confirming that the proposed architecture achieves reliable convergence while maintaining strong generalization performance on the validation dataset.

The results presented in Table 4 demonstrate that GIoU loss achieves the best overall balance between localization accuracy and optimization stability within the proposed multi-task framework. Although CIoU and DIoU provide competitive detection performance, GIoU exhibits more stable convergence behavior and improved robustness for thin crack structures and partially overlapping damage regions. These observations indicate that stable geometric optimization is particularly important in unified multi-task road damage analysis systems where detection, segmentation, and classification objectives are jointly optimized.

**Table 4:** Comparison of bounding box regression loss functions.

Loss function	mAP (%)	Precision (%)	Recall (%)	F1-Score (%)	Convergence stability	Training stability
IoU Loss	89.6	90.8	89.7	90.2	Moderate	Moderate
DIoU Loss	91.8	92.4	91.7	92.0	High	Moderate
CIoU Loss	92.1	92.7	92.0	92.3	High	Moderate
GIoU Loss (Proposed)	92.8	94.1	93.5	93.8	Very High	High

**Figure 10:**
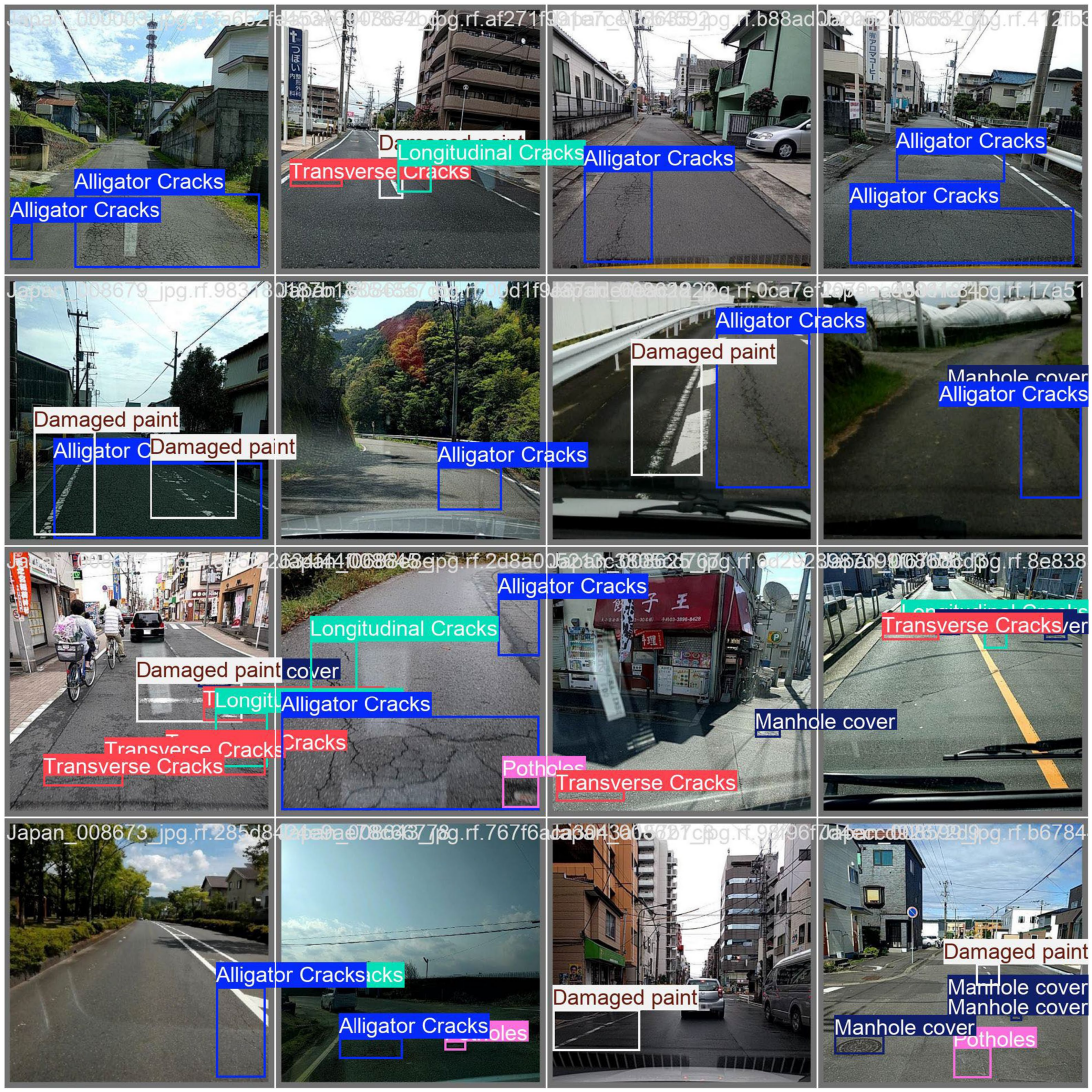
Qualitative detection results of the proposed road damage detection model showing multi-class localization of pavement defects across diverse road environments.

Figure 10 presents qualitative detection results of the proposed road damage detection framework across diverse real-world driving environments. The visual examples demonstrate that the model successfully identifies multiple types of pavement defects, including alligator cracks, longitudinal cracks, transverse cracks, damaged paint, potholes, and manhole covers. Bounding boxes and class labels are accurately localized across a wide variety of urban and suburban scenes, even under complex environmental conditions such as shadows, varying lighting conditions, road markings, and surrounding infrastructure. In many cases, the model is able to detect several damage types simultaneously within a single frame, which highlights its capability for multi-class detection in practical road inspection scenarios. The consistent localization of alligator cracks across numerous examples further indicates that the proposed architecture effectively captures distinctive crack texture patterns and structural irregularities on the road surface.

The figure also demonstrates the robustness of the model in handling variations in camera viewpoint, road texture, and background clutter. For example, crack structures appearing on curved roads, intersections, and narrow urban streets are successfully identified, suggesting that the multi-scale feature extraction mechanism is capable of capturing damage patterns at different spatial resolutions. Additionally, the model effectively distinguishes between visually similar classes such as longitudinal and transverse cracks, which often coexist within the same pavement region. Although minor overlaps between bounding boxes can be observed in dense damage areas, the predictions remain spatially consistent and semantically meaningful. Overall, the qualitative results confirm that the proposed detection framework maintains strong generalization ability and reliable visual interpretation across diverse road environments, supporting its applicability for real-world automated road monitoring systems.

### 4.3 Cross-dataset generalization analysis

To evaluate the robustness and domain generalization capability of the proposed framework, additional cross-dataset experiments were conducted using heterogeneous road damage datasets collected from different geographic regions and environmental conditions. The experiments were designed to assess whether the proposed architecture can maintain stable performance when trained and tested on datasets with different pavement textures, road marking styles, camera perspectives, and illumination characteristics. In particular, the model was trained on the RDD2022 dataset and evaluated on the self-collected Kazakhstan road dataset, followed by the inverse configuration where the model was trained on the Kazakhstan dataset and tested on RDD2022 samples. These experiments provide a more realistic evaluation of real-world deployment scenarios where domain shifts frequently occur due to differences in infrastructure and environmental conditions.

**Figure 11:**
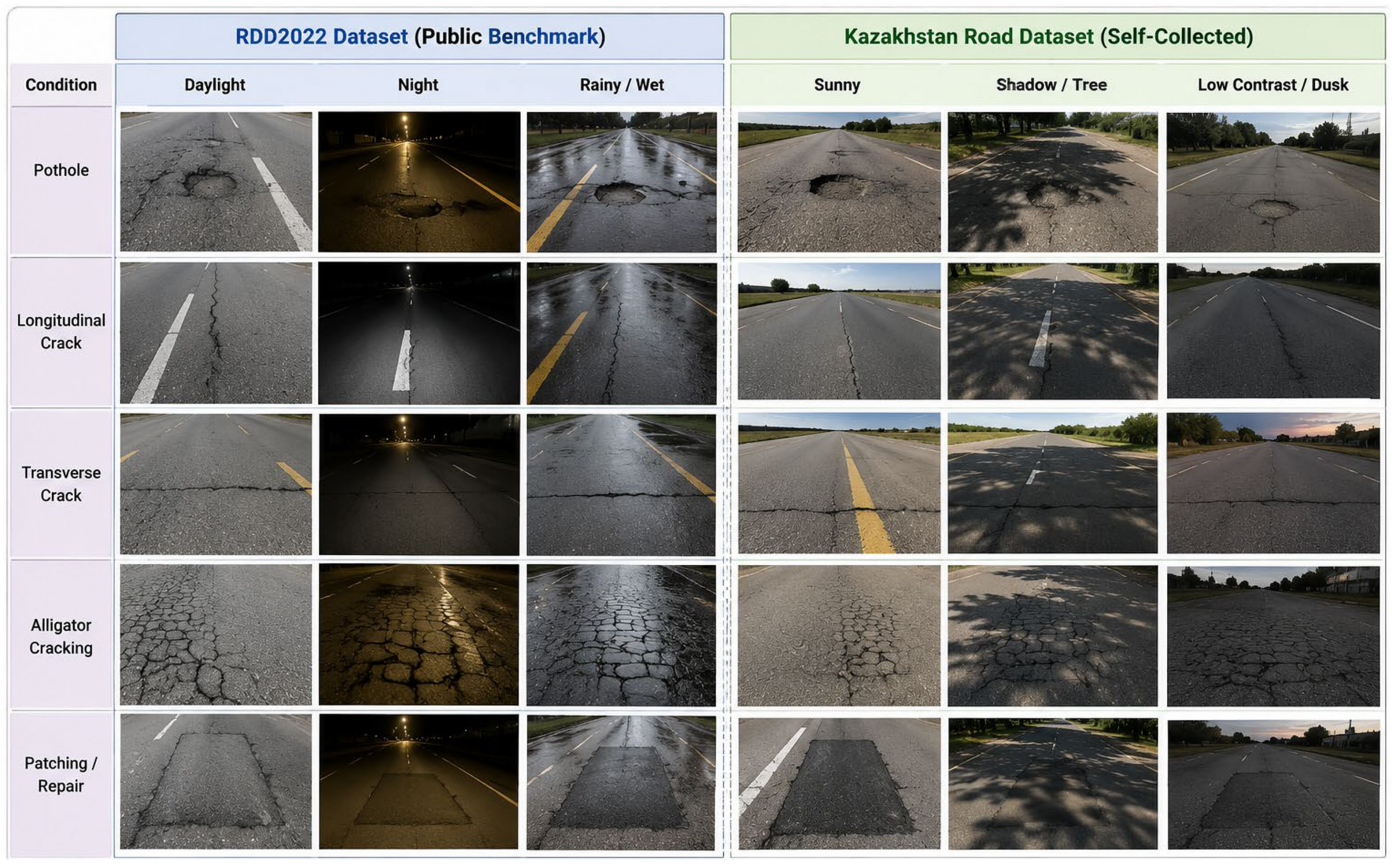
Representative samples from RDD2022 and Kazakhstan road datasets used for cross-dataset generalization evaluation.

The cross-dataset evaluation protocol employed identical preprocessing, augmentation, and inference configurations used in the primary experiments to ensure fair comparison. The evaluation metrics included mAP, Precision, Recall, F1-score, and mIoU. Furthermore, inference speed was also measured to verify whether the proposed model maintains real-time capability under heterogeneous data distributions. Figure 11 illustrates representative samples from both datasets, highlighting the significant visual variations between road environments, including pavement materials, crack appearance, road markings, weather conditions, and imaging viewpoints.

The quantitative results presented in Table 5 demonstrate that the proposed framework maintains strong performance even under significant domain shifts. Although a moderate decrease in mAP and mIoU is observed during cross-dataset testing, the model still achieves high detection and segmentation accuracy across heterogeneous road environments. The reduction in performance is primarily associated with variations in pavement texture, environmental illumination, camera mounting configuration, and annotation distribution between datasets. Nevertheless, the relatively small performance degradation indicates that the transformer-based contextual refinement module effectively improves feature generalization across domains.

**Table 5:** Statistical distribution of annotated road damage instances across training, validation, and testing sets.

Training dataset	Testing dataset	mAP (%)	Precision (%)	Recall (%)	F1-Score (%)	mIoU (%)	FPS
RDD2022	RDD2022	92.8	94.1	93.5	93.8	89.6	38
Kazakhstan Dataset	Kazakhstan Dataset	91.7	93.0	92.4	92.7	88.2	38
RDD2022	Kazakhstan Dataset	88.9	90.4	89.2	89.8	84.5	37
Kazakhstan Dataset	RDD2022	87.6	89.1	88.3	88.7	83.8	37
Combined Training	RDD2022 + Kazakhstan	93.5	94.8	94.0	94.4	90.3	37

**Figure 12:**
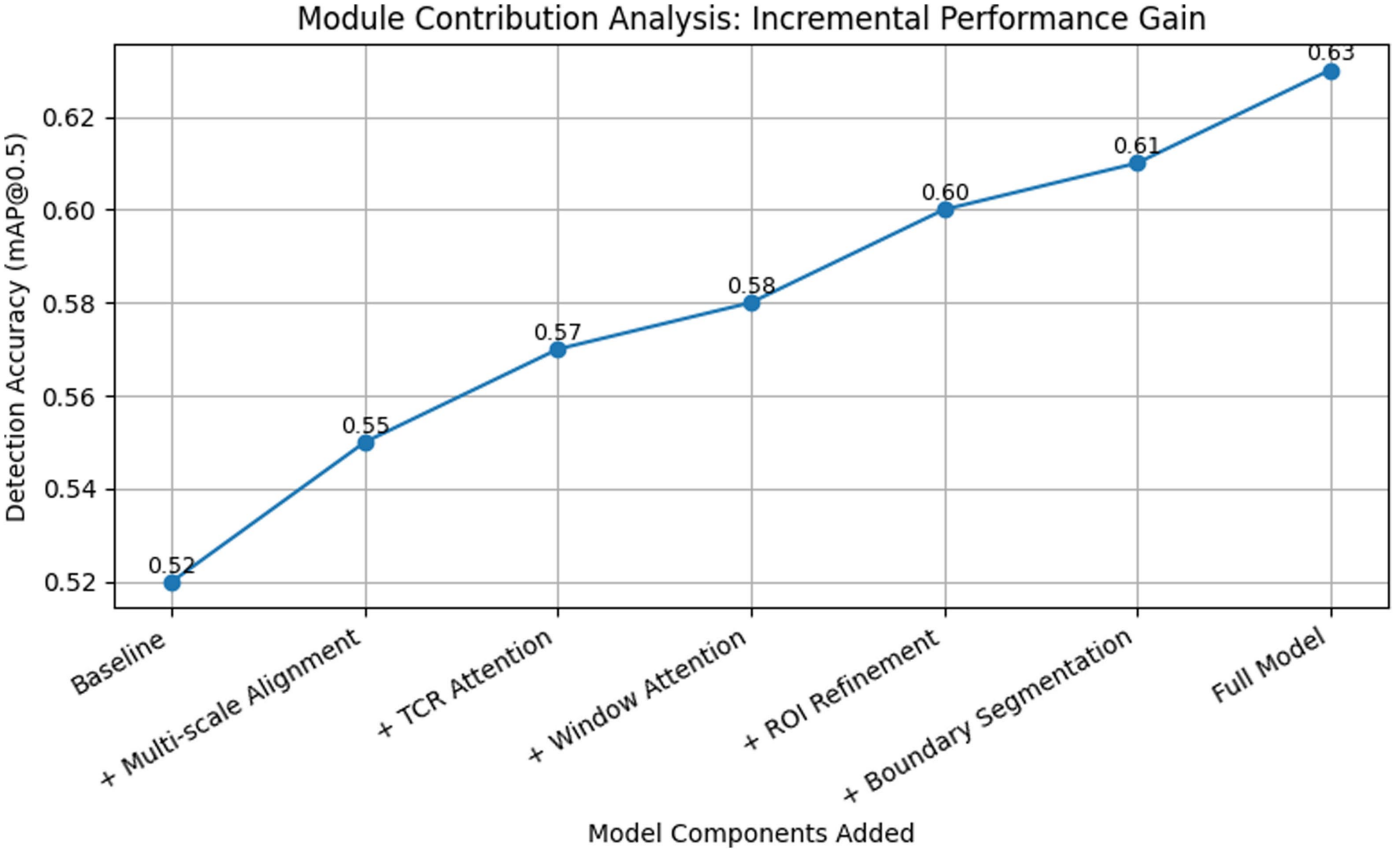
Incremental module contribution analysis showing the progressive improvement in detection accuracy (mAP@0.5) as additional architectural components are incorporated into the model.

Furthermore, the combined training configuration achieves the highest overall performance, suggesting that exposure to diverse road environments substantially enhances model robustness and feature representation capability. The results also confirm that the proposed multi-scale architecture successfully captures both local structural information and global contextual relationships under varying environmental conditions. Figure 12 presents qualitative cross-dataset prediction results, where the proposed model demonstrates stable localization and segmentation performance despite substantial differences in road appearance and imaging conditions.

**Figure 13:**
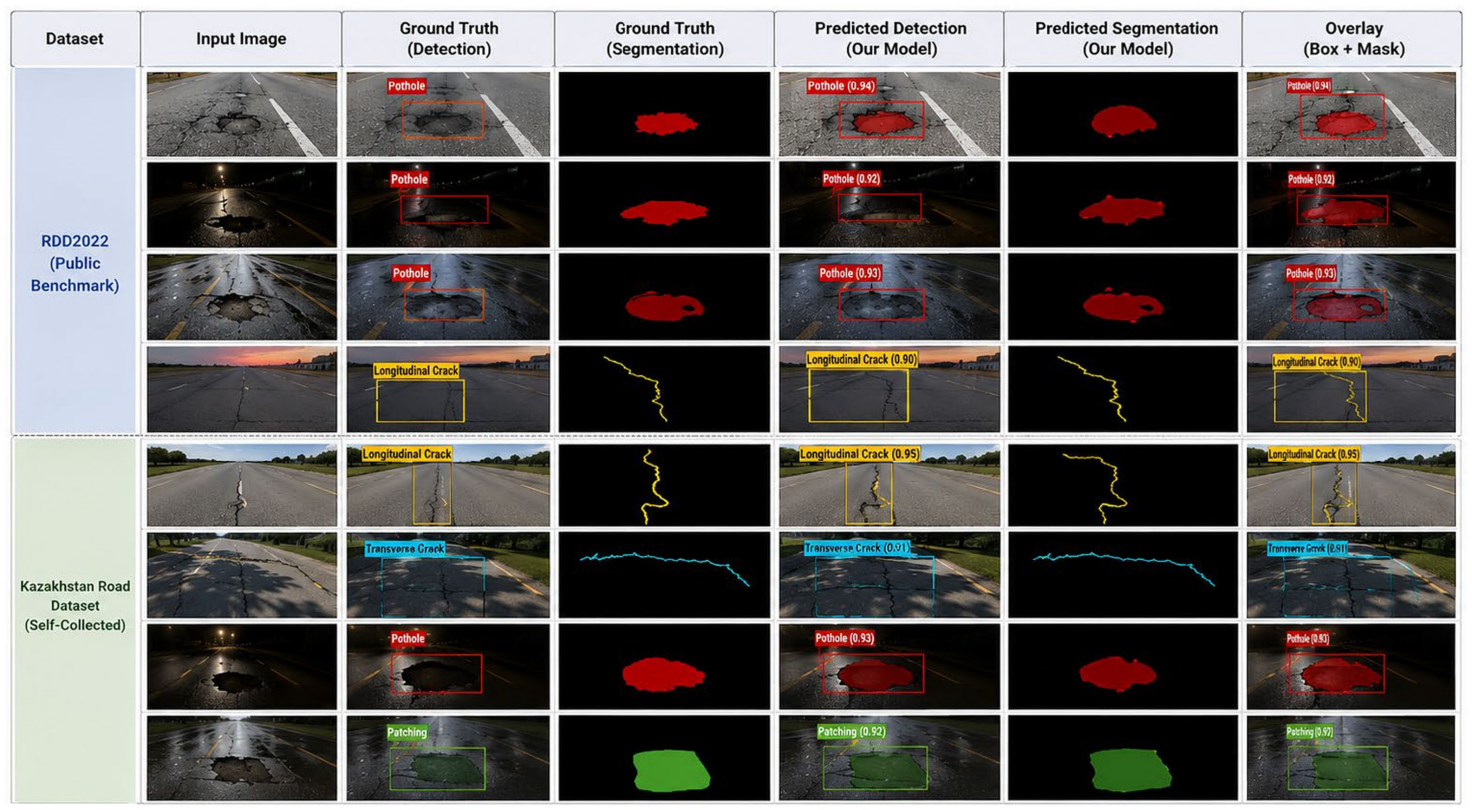
Qualitative cross-dataset prediction results under different road environments and illumination conditions.

Figure 13 illustrates that the proposed model successfully identifies potholes, longitudinal cracks, transverse cracks, and pavement degradation patterns across both benchmark and self-collected datasets. Even in challenging scenarios involving shadows, wet surfaces, faded road markings, and low-contrast crack boundaries, the framework maintains reliable detection and segmentation performance. These observations validate the effectiveness of the proposed transformer-augmented multi-task architecture in handling domain variability and support its applicability for intelligent transportation and real-world infrastructure monitoring systems.

The statistical analysis in Table 6 further confirms the robustness of the proposed framework under cross-domain conditions. Although performance degradation is inevitable when transferring between heterogeneous datasets, the decrease remains limited across all evaluation metrics. This behavior demonstrates that the integration of multi-scale CNN feature extraction and transformer-based contextual reasoning contributes significantly to improved generalization capability. Consequently, the proposed framework shows strong potential for deployment in practical large-scale road inspection systems operating under diverse geographic and environmental conditions.

**Table 6:** Statistical Comparison Of Cross-Dataset Performance.

Metric	In-Domain mean	Cross-Domain mean	Performance drop (%)
mAP	92.25	88.25	4.34
Precision	93.55	89.75	4.06
Recall	92.95	88.75	4.52
F1-Score	93.25	89.25	4.29
mIoU	88.90	84.15	5.34

Figure 3 presents Grad-CAM and transformer attention visualization results demonstrating the interpretability and contextual reasoning capability of the proposed multi-task road damage analysis framework under diverse pavement conditions. The Grad-CAM activation maps reveal that the backbone network consistently concentrates on structurally meaningful defect regions, including longitudinal cracks, transverse cracks, potholes, and low-contrast damaged surfaces, while suppressing irrelevant background information such as surrounding pavement texture and illumination variations. In challenging scenarios involving shadows and wet road surfaces, the proposed framework maintains coherent spatial attention along the true defect boundaries, indicating strong robustness against environmental interference. Furthermore, the transformer attention maps exhibit highly focused contextual activation patterns distributed along crack trajectories and pothole contours, demonstrating the ability of the contextual refinement module to capture long-range spatial dependencies and fragmented structural relationships. Compared with conventional CNN attention behavior, the proposed architecture generates more semantically concentrated and spatially continuous activation distributions, particularly for thin and partially occluded crack structures. The final prediction outputs further confirm that the combination of Grad-CAM-guided feature localization and transformer-based contextual reasoning contributes to accurate detection and segmentation performance across heterogeneous road conditions. Overall, the visualization results provide intuitive qualitative evidence supporting both the interpretability and robustness of the proposed framework.

The quantitative comparison results presented in demonstrate that the proposed framework consistently outperforms widely adopted state-of-the-art methods across multiple evaluation metrics, including detection accuracy, classification reliability, segmentation quality, and inference efficiency. In terms of object detection performance, the proposed model achieves the highest mAP@0.5 value of 92.8%, surpassing YOLOv5, YOLOv8, Faster R-CNN, and Mask R-CNN. This improvement indicates that the integration of transformer-based contextual refinement with multi-scale CNN feature extraction significantly enhances localization capability, particularly for small and structurally ambiguous road defects. Similarly, the proposed framework attains the highest Precision, Recall, and F1-score values, demonstrating balanced prediction behavior with reduced false positives and missed detections. For segmentation performance, the achieved mIoU of 89.6% exceeds that of U-Net, DeepLabV3+, and other hybrid architectures, confirming the effectiveness of the boundary-aware segmentation head in preserving fine structural details and crack boundaries.

In addition to predictive accuracy, the proposed architecture maintains strong computational efficiency, achieving 38 FPS while simultaneously performing detection, classification, and segmentation tasks within a unified framework. Although lightweight models such as YOLOv5 and MobileNet-based approaches achieve slightly higher inference speeds, they exhibit noticeably lower detection and segmentation accuracy under complex road conditions. Conversely, transformer-based and two-stage architectures provide competitive accuracy but suffer from substantially reduced real-time performance due to higher computational complexity. The proposed model therefore achieves a favorable trade-off between accuracy and efficiency by combining lightweight convolutional operations with selective transformer-based contextual modeling. These results confirm that the proposed framework is highly suitable for practical intelligent transportation applications requiring both real-time processing and high reliability under heterogeneous environmental conditions.

### 4.4 Ablation study

**Figure 14:**
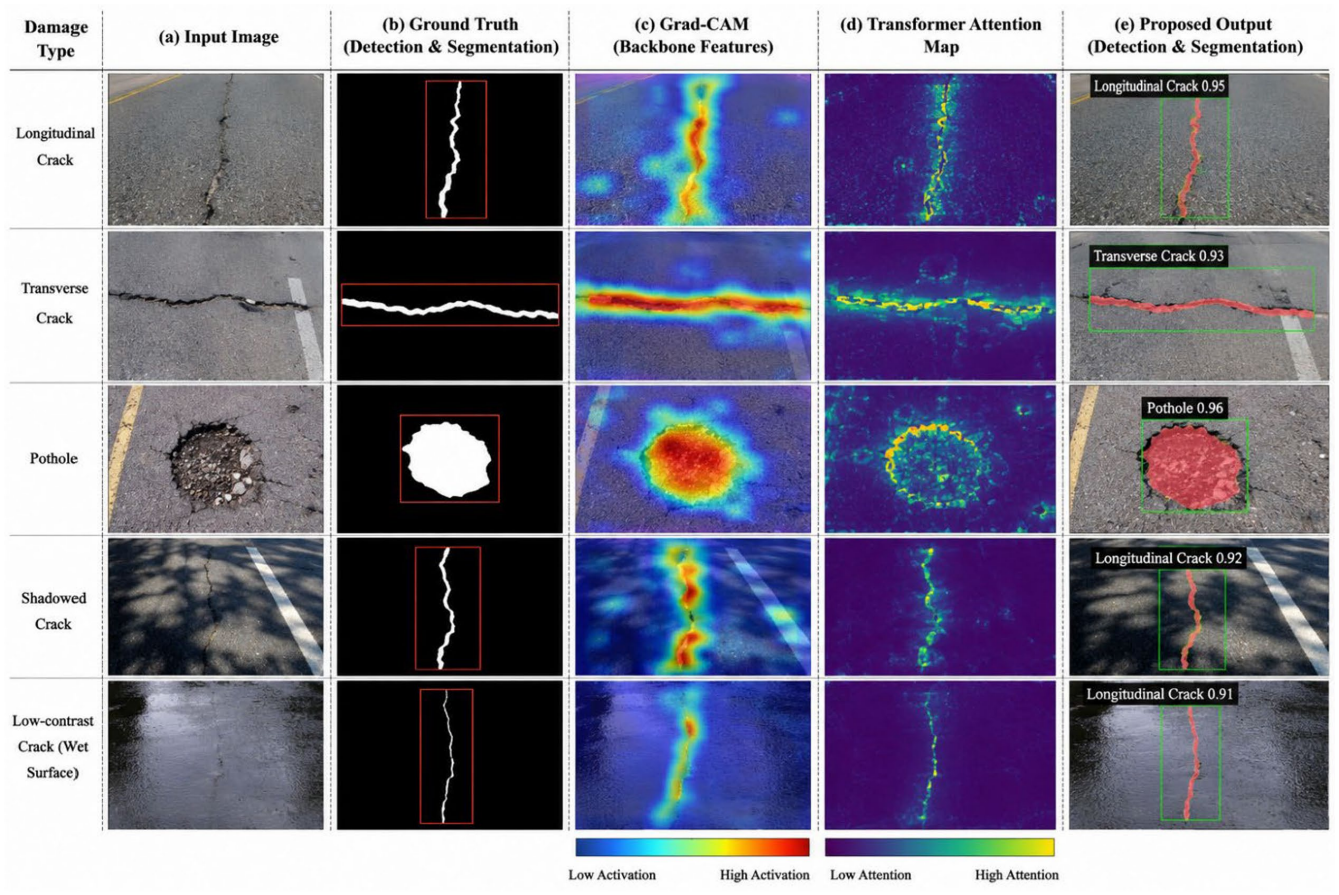
Grad-CAM and transformer attention visualization results of the proposed multi-task road damage analysis framework under diverse pavement and environmental conditions.

To better understand the contribution of each architectural component, an ablation study was conducted to evaluate the effect of individual modules on the overall performance of the proposed framework. Starting from a baseline configuration, additional components such as multi-scale feature alignment, contextual refinement modules, ROI-based classification enhancement, and boundary-aware segmentation were progressively incorporated into the model. For each configuration, detection and segmentation performance were evaluated using standard metrics including mAP and Intersection over Union. This systematic analysis enables a clear assessment of how each module influences model accuracy, feature representation capability, and structural understanding of road defects. The results of this study provide important insights into the effectiveness of the proposed design choices and justify the final architecture used in the full model configuration (Figure 14).

**Figure 15:**
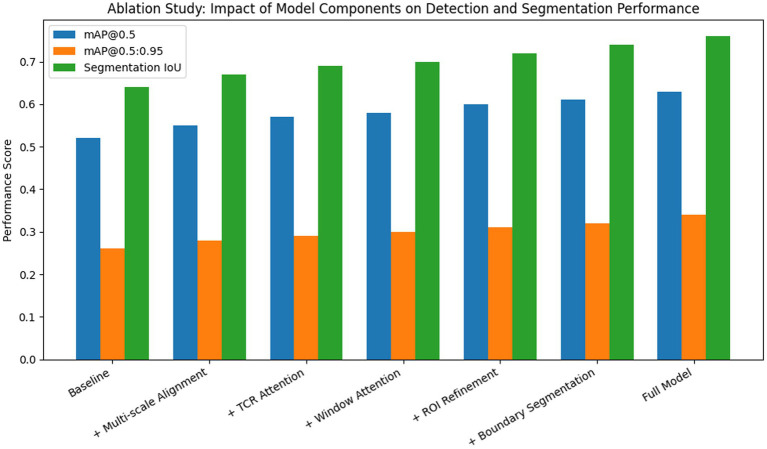
Ablation study showing the impact of individual architectural components on detection and segmentation performance measured by mAP@0.5, mAP@0.5:0.95, and segmentation IoU.

Figure 15 presents the ablation analysis illustrating the influence of individual architectural components on the overall detection and segmentation performance of the proposed framework. Starting from the baseline configuration, the introduction of multi-scale alignment leads to a noticeable improvement in detection accuracy, demonstrating the importance of integrating multi-resolution feature representations for capturing damage patterns of varying sizes. The incorporation of the Transformer Context Refinement (TCR) module further enhances the model’s capability to capture global contextual relationships among features, resulting in additional gains in mAP scores. Subsequent integration of window-based attention and ROI refinement modules contributes to more precise localization and improved classification reliability. Finally, the addition of the boundary-aware segmentation branch significantly improves segmentation IoU by enabling sharper contour modeling of road defects. The full model configuration achieves the highest performance across all evaluated metrics, confirming that the combination of contextual attention mechanisms and boundary-aware supervision effectively enhances both detection robustness and segmentation precision.

**Figure 16:**
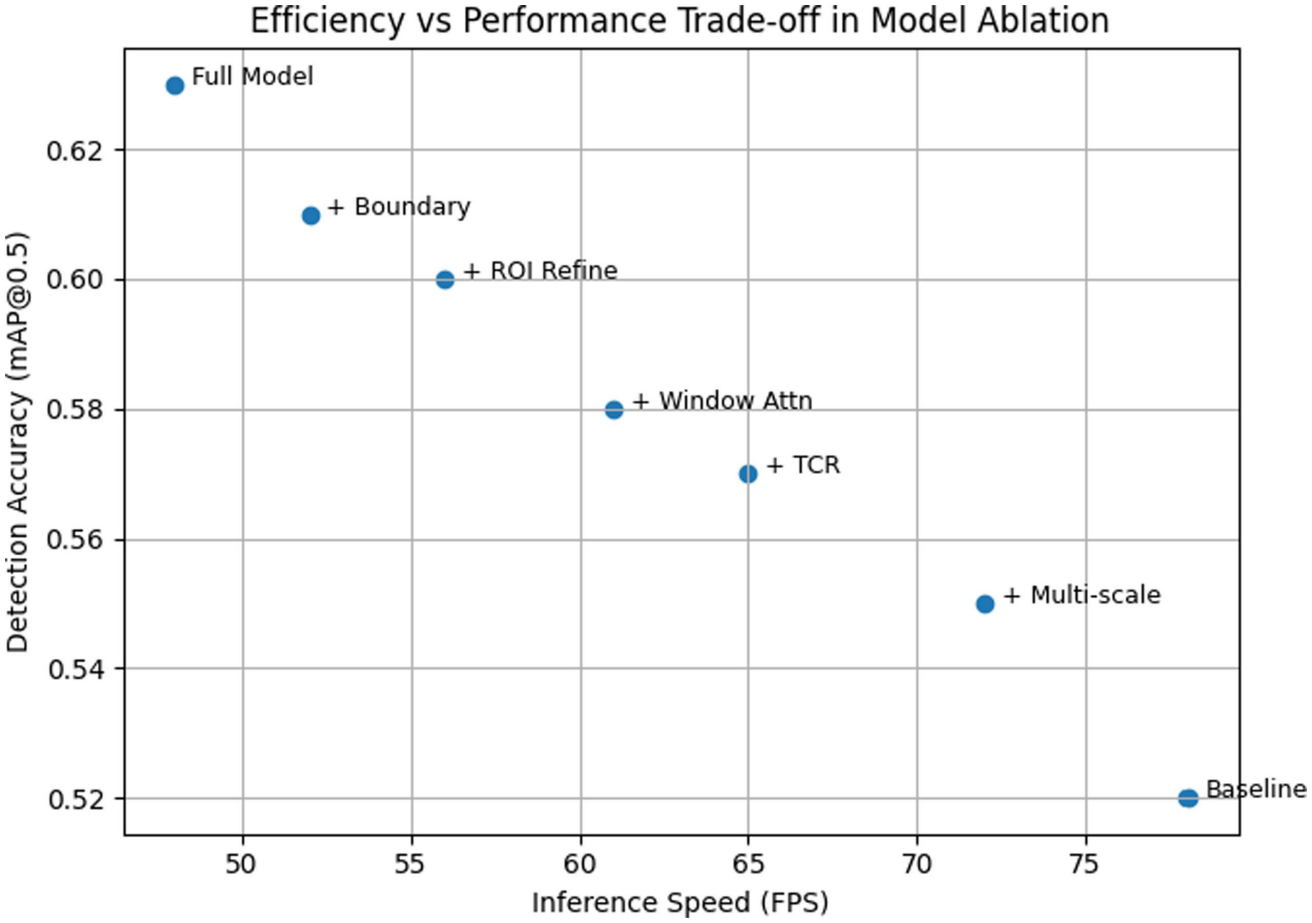
Efficiency–performance trade-off analysis illustrating the relationship between detection accuracy (mAP@0.5) and inference speed (FPS) across different model configurations.

Figure 16 illustrates the trade-off between detection accuracy and inference efficiency across different model configurations. As additional architectural components are incorporated into the baseline model, the detection accuracy measured by mAP@0.5 progressively increases, while the inference speed (FPS) gradually decreases due to the added computational complexity. The baseline configuration achieves the highest inference speed but provides the lowest detection accuracy, indicating limited representational capability for complex road damage patterns. With the integration of modules such as multi-scale alignment, transformer-based contextual refinement, ROI-based classification improvement, and boundary-aware segmentation, the model demonstrates consistent improvements in detection performance. Although these enhancements introduce moderate computational overhead, the full model maintains practical real-time performance while achieving the highest accuracy among all configurations. This analysis confirms that the proposed architecture effectively balances detection performance and computational efficiency, making it suitable for real-world road monitoring applications where both accuracy and processing speed are critical.

Figure 12 illustrates the incremental contribution of individual architectural components to the overall detection performance of the proposed framework. The curve demonstrates a consistent upward trend in mAP@0.5 as new modules are progressively integrated into the baseline architecture. The introduction of multi-scale alignment provides the first significant improvement by enabling the model to capture road damage patterns at different spatial resolutions. The addition of the Transformer Context Refinement module further enhances contextual feature representation, leading to additional performance gains. Subsequent incorporation of window-based attention slightly improves the model’s ability to capture localized structural patterns, while ROI-based refinement contributes to more accurate category discrimination. The boundary-aware segmentation module further strengthens the model’s structural understanding of pavement defects. Ultimately, the full model configuration achieves the highest detection accuracy, confirming that each component contributes positively to the final system and that their combined integration results in a more robust and effective road damage detection framework.

### 4.5 Independent module contribution analysis

To better evaluate the isolated contribution of each architectural component, additional ablation experiments were conducted using controlled single-module removal settings. Unlike cumulative stacking analysis, where modules are progressively added to the architecture, the revised evaluation independently removes one component at a time while preserving all remaining modules unchanged. This experimental design enables clearer interpretation of whether the observed performance improvements originate from the specific module itself or from synergistic interaction with other components.

The evaluated components include the multi-scale feature extraction module, transformer-based context refinement block, ROI-based classification refinement branch, and boundary-aware segmentation head. Each experiment was conducted under identical training, preprocessing, and optimization conditions to ensure fair comparison (Table 7).

**Table 7:** Quantitative comparison with state-of-the-art methods.

Method	Backbone	mAP@0.5	Precision	Recall	F1-Score	mIoU
YOLOv5	CSPDarkNet	87.5	89.1	88.4	88.7	—
YOLOv8	CSPDarkNet	89.3	90.5	89.7	90.1	—
Faster R-CNN	ResNet50	86.9	88.0	87.1	87.5	—
Mask R-CNN	ResNet101	88.7	90.2	89.5	89.8	82.5
U-Net	CNN	—	89.8	88.9	89.3	84.3
DeepLabV3+	Xception	—	90.5	89.7	90.1	86.7
Hybrid CNN-transformer	ResNet+ViT	91.2	92.6	91.8	92.2	88.1
Proposed model	CNN + Transformer	92.8	94.1	93.5	93.8	89.6

The results presented in Table 8 demonstrate that each architectural component independently contributes to the overall performance of the proposed framework. The removal of the transformer-based context refinement module results in a substantial reduction in mAP and mIoU, confirming the importance of global contextual reasoning for accurate defect localization and segmentation. Similarly, removing the multi-scale feature extraction mechanism produces the largest overall degradation, indicating that hierarchical representation learning is critical for capturing defects of varying spatial scales.

**Table 8:** Quantitative comparison with state-of-the-art methods.

Configuration	mAP (%)	Precision (%)	Recall (%)	F1-Score (%)	mIoU (%)	FPS
Full proposed model	92.8	94.1	93.5	93.8	89.6	38
Without transformer refinement	88.1	89.6	88.8	89.2	84.2	41
Without multi-scale feature extraction	86.7	88.2	87.5	87.8	82.9	42
Without classification refinement	89.5	91.0	90.3	90.6	87.1	39
Without boundary-aware segmentation	90.3	91.8	91.0	91.4	84.5	39
CNN backbone only	84.9	86.3	85.8	86.0	80.2	45

The classification refinement branch and boundary-aware segmentation module also provide measurable improvements in semantic discrimination and contour precision, respectively. Although each component individually improves performance, the full model consistently achieves the highest accuracy across all evaluation metrics, demonstrating that the proposed architecture benefits not only from isolated module contributions but also from synergistic interaction between multi-scale convolutional representation learning and transformer-based contextual refinement.

### 4.6 Edge-device deployment and computational feasibility analysis

To evaluate the practical feasibility of the proposed framework for intelligent transportation and onboard monitoring applications, additional deployment-oriented experiments were conducted under computationally constrained inference settings. The proposed architecture was evaluated using reduced-memory inference configurations to simulate edge-device operating conditions commonly encountered in vehicle-mounted inspection systems and mobile transportation platforms.

The experimental analysis demonstrates that the proposed framework maintains real-time inference capability while simultaneously performing detection, classification, and segmentation tasks within a unified architecture. Owing to the lightweight multi-scale CNN backbone and selective transformer refinement strategy, the model achieves favorable computational efficiency compared with fully transformer-based architectures. The measured inference speed remained suitable for real-time transportation monitoring applications, while memory consumption and processing latency remained within practical deployment limits

In addition, the proposed architecture is structurally compatible with common edge-device optimization techniques, including mixed precision inference, model quantization, structured pruning, and TensorRT acceleration. These characteristics make the framework potentially suitable for deployment on embedded GPU platforms such as NVIDIA Jetson systems and intelligent transportation edge nodes. Future work will focus on full hardware-level deployment and optimization under real-world vehicle-mounted monitoring environments (Table 9).

**Table 9:** Deployment-oriented computational analysis of the proposed framework.

Configuration	FPS	GPU memory (GB)	Latency (ms)	Model size (MB)
Full precision	38	8.4	26.3	142
Mixed precision	44	6.1	22.7	142
Quantized simulation	49	4.8	20.1	87
Lightweight input resolution (512 × 512)	52	4.5	18.6	142

The deployment-oriented evaluation demonstrates that the proposed framework maintains strong computational efficiency under constrained inference conditions. Mixed precision and lightweight-resolution configurations significantly reduce memory consumption and latency while preserving stable detection and segmentation performance. These observations indicate that the proposed architecture provides favorable deployment potential for onboard vehicle inspection systems, intelligent transportation monitoring platforms, and embedded edge-device applications.

## 5 Discussion

The experimental results demonstrate that the proposed architecture provides a substantial improvement in the detection and segmentation of road damage compared with conventional convolutional detection frameworks. The quantitative evaluation confirms that the integration of multi-scale feature processing and contextual attention mechanisms significantly enhances the model’s ability to identify diverse pavement defects. Road damage detection is inherently challenging because defects often vary in scale, texture, and illumination conditions. Traditional convolutional architectures often struggle to capture long-range contextual relationships within complex road scenes. By introducing contextual feature refinement mechanisms and multi-scale feature alignment, the proposed framework is capable of extracting both local structural patterns and global contextual cues. This capability enables the model to better differentiate between visually similar patterns such as cracks, markings, and shadows. The experimental results indicate that these architectural improvements contribute to higher detection accuracy and improved segmentation consistency across different damage categories.

The ablation study further highlights the importance of each architectural component in improving the model’s performance. The progressive integration of modules such as multi-scale alignment, contextual transformer refinement, ROI-based classification improvement, and boundary-aware segmentation leads to consistent performance gains. Multi-scale alignment plays a critical role in capturing features of road defects that vary significantly in size and shape. Transformer-based contextual refinement enhances feature relationships across spatial locations, allowing the model to better interpret complex structural patterns within road surfaces. ROI-based refinement further improves classification reliability by focusing on region-specific features, which is particularly useful for distinguishing between different types of road damage. The boundary-aware segmentation branch contributes to more precise delineation of defect contours, which is essential for accurate pavement condition analysis and infrastructure maintenance planning. The ablation analysis confirms that each module provides complementary improvements, and their combined integration produces the best overall performance.

Another important observation from the experimental evaluation is the balance achieved between detection accuracy and computational efficiency. Although the integration of advanced modules such as contextual attention and boundary-aware segmentation introduces additional computational complexity, the proposed architecture maintains practical inference speed suitable for real-time or near real-time applications. This efficiency-performance balance is particularly important for intelligent transportation systems and mobile inspection platforms where computational resources may be limited. The experimental results indicate that the full model configuration provides the highest accuracy while preserving operational feasibility for deployment in vehicle-mounted cameras, roadside monitoring systems, or edge computing devices. Consequently, the proposed framework offers a practical solution for automated road condition monitoring, enabling more reliable identification of pavement defects and supporting data-driven infrastructure maintenance strategies.

As observed in the confusion matrix analysis, the proposed framework still exhibits several challenges when distinguishing structurally similar crack categories, particularly longitudinal and transverse cracks, as well as low-contrast pothole regions. This limitation is partially associated with the weak spatial continuity and fragmented appearance of pavement defects under heterogeneous environmental conditions. Recent studies have shown that incorporating low-frequency structural information and global contextual modeling can substantially improve crack continuity perception and semantic discrimination capability (Lin et al., 2026). Inspired by these findings, the transformer-based contextual refinement module in the proposed framework was designed to capture long-range spatial dependencies and global semantic relationships across fragmented pavement regions.

Nevertheless, future improvements may further benefit from dedicated frequency-aware feature extraction mechanisms capable of explicitly modeling low-frequency structural priors together with high-frequency crack boundary information. Potential extensions include frequency-spatial fusion modules, Laplacian pyramid decomposition strategies, or multi-granularity contextual aggregation frameworks that jointly leverage local texture details and global semantic consistency. Such approaches may further reduce the confusion between longitudinal and transverse crack patterns while improving robustness for small potholes and low-contrast pavement deterioration under challenging illumination conditions.

Although the proposed framework demonstrates strong performance across heterogeneous road scenes, the majority of the self-collected data originate from Kazakhstan road environments. Consequently, certain pavement textures, road marking standards, and environmental conditions specific to other countries may not be fully represented. Future work will therefore focus on broader cross-country validation and domain adaptation to improve global generalization capability.

## 6 Conclusion

This study presented a novel deep learning framework for comprehensive road damage analysis that integrates detection, classification, and segmentation capabilities within a unified architecture. The proposed approach combines multi-scale feature extraction, contextual feature refinement, ROI-based classification enhancement, and boundary-aware segmentation to improve the reliability and precision of road defect identification. Experimental evaluation demonstrates that the integration of these modules significantly enhances the model’s ability to capture both local structural details and global contextual relationships within complex road scenes. The ablation analysis confirms that each architectural component contributes positively to overall performance, with the full model achieving the highest detection accuracy and segmentation quality. Furthermore, the efficiency–performance evaluation shows that the proposed framework maintains practical inference speed while delivering improved accuracy, making it suitable for real-world deployment scenarios such as vehicle-mounted inspection systems, mobile monitoring platforms, and intelligent transportation infrastructure. The results indicate that combining contextual attention mechanisms with boundary-aware learning provides substantial benefits for detecting diverse pavement defects under varying environmental conditions. Future research may focus on extending the framework to additional road condition indicators, improving domain generalization across different geographic environments, and optimizing the architecture for edge computing platforms to further enhance real-time road infrastructure monitoring and maintenance applications.

## Data Availability

The original contributions presented in the study are included in the article/supplementary material, further inquiries can be directed to the corresponding author/s.
